# Integrated Transcriptomics and Metabolomics Analyses Provide Insights Into the Response of Chongyi Wild Mandarin to *Candidatus* Liberibacter Asiaticus Infection

**DOI:** 10.3389/fpls.2021.748209

**Published:** 2021-10-14

**Authors:** Ting Peng, Jing-Liang Kang, Xin-Ting Xiong, Fang-Ting Cheng, Xiao-Juan Zhou, Wen-Shan Dai, Min Wang, Zhong-Yang Li, Hua-Nan Su, Ba-Lian Zhong

**Affiliations:** ^1^National Navel Orange Engineering Research Center, College of Life Sciences, Gannan Normal University, Ganzhou, China; ^2^China-USA Citrus Huanglongbing Joint Laboratory, Ganzhou, China

**Keywords:** Huanglongbing, wild citrus germplasm, RNA-seq, metabolome, tissue specificity, anatomical aberration

## Abstract

*Candidatus* Liberibacter asiaticus (*C*Las) is the causative agent of Huanglongbing (HLB), which has caused great economic losses to the citrus industry. The molecular mechanism of the host response to *C*Las in wild citrus germplasm has been reported less. Eighteen weeks after inoculation *via* grafting, all the *C*Las-inoculated Chongyi wild mandarin (*Citrus reticulata*) were positive and showed severe anatomical aberrations, suggesting its susceptibility to HLB. Transcriptomics and metabolomics analyses of leaves, barks, and roots from mock-inoculated (control) and *C*Las-inoculated seedlings were performed. Comparative transcriptomics identified 3,628, 3,770, and 1,716 differentially expressed genes (DEGs) between *C*Las-infected and healthy tissues in the leaves, barks, and roots, respectively. The *C*Las-infected tissues had higher transcripts per kilobase per million values and more genes that reached their maximal expression, suggesting that HLB might cause an overall increase in transcript accumulation. However, HLB-triggered transcriptional alteration showed tissue specificity. In the *C*Las-infected leaves, many DEGs encoding immune receptors were downregulated. In the *C*Las-infected barks, nearly all the DEGs involved in signaling and plant-pathogen interaction were upregulated. In the *C*Las-infected roots, DEGs encoding enzymes or transporters involved in carotenoid biosynthesis and nitrogen metabolism were downregulated. Metabolomics identified 71, 62, and 50 differentially accumulated metabolites (DAMs) in the *C*Las-infected leaves, barks and roots, respectively. By associating DEGs with DAMs, nitrogen metabolism was the only pathway shared by the three infected tissues and was depressed in the *C*Las-infected roots. In addition, 26 genes were determined as putative markers of *C*Las infection, and a hypothesized model for the HLB susceptibility mechanism in Chongyi was proposed. Our study may shed light on investigating the molecular mechanism of the host response to *C*Las infection in wild citrus germplasm.

## Introduction

Also known as citrus greening disease, Huanglongbing (HLB) is one of the most destructive diseases of citrus and has resulted in huge economic losses to the citrus industry. It is caused by the phloem-limited, gram-negative, alpha-proteobacteria *Candidatus* Liberibacter spp. [i.e., *C*. L. asiaticus (*C*Las), *C*. L. africanus (*C*Laf), and *C*. L. americanus (*C*Lam)] (Albrecht and Bowman, 2008). *C*Las is transmitted *via* grafting with *C*Las-infected scions and phloem-feeding psyllids [Asian citrus psyllid (ACP) *Diaphorina citri*] (Bové, 2006). Once infected, the anatomical aberration in newly growing flushes was observed at the early invasion of the pathogen (Folimonova and Achor, 2010). As HLB develops, infected citrus plants will manifest a series of typical symptoms, such as leaves with an asymmetrical pattern of blotchy yellowing or mottling and malformed fruits with aborted seeds (Fu et al., 2016). Eventually, *C*Las can reduce resource availability for roots and then leads to starvation, which results in widespread tree death due to the decreased photoassimilate transport (Wang and Trivedi, 2013).

To investigate the underlying pathogenetic mechanism of HLB, studies have scanned changes in gene expression levels in *C*Las-infected citrus (Wang et al., 2016; Rawat et al., 2017; Zhao et al., 2019). In *C*Las-infected leaves, many differentially expressed genes (DEGs) involved in carbohydrate metabolism, particularly starch pathways, were found to be significantly upregulated (Albrecht and Bowman, 2008). A study performed on citrus phloem by microarray revealed that HLB could disrupt various biological functions, such as sugar metabolism, plant defense, phytohormones, and cell wall metabolism (Kim et al., 2009). The transcriptome of the *C*Las-positive citrus roots showed that HLB could alter some biological processes, such as cell wall modifications, protease-involved protein degradation, carbohydrate metabolism, hormone synthesis and signaling, transcription activities, and stress responses (Zhong et al., 2015). Nevertheless, there are rare reports on the comparison of transcript differences among various *C*Las-positive tissues, especially in wild citrus germplasms.

Moreover, bioinformatic analysis of four RNA-Seq datasets on HLB response and tolerance in leaves revealed that sucrose and starch metabolism was highly linked with disease symptoms, but that DEGs in each data set varied because of the conditions of leaves, infection stages, and environments (Balan et al., 2018). Therefore, analysis on transcriptional level alone might be difficult to screen the critical molecular signals in response to *C*Las infection. Metabolomics could identify low-molecular-weight metabolites (<1 kDa) that alter their accumulation in response to physiological challenges (Padhi et al., 2019; Huang et al., 2020). Integrated transcriptomics and metabolomics analysis may identify key HLB-related pathways, in which DEGs and differentially accumulated metabolites (DAMs) are both enriched.

To evaluate the influences of HLB on various tissues, RNA-seq and metabolome libraries were constructed with leaves, barks, and roots from healthy and *C*Las-infected Chongyi wild mandarin (*Citrus reticulata*), wild citrus germplasm in Jiangxi province, China (Peng et al., 2021). Based on the number of reads per gene, the number of DEGs between *C*Las-infected vs. healthy leaves/barks/roots was estimated, and their putative functions were investigated. Parallel gene expression changes among tissues were compared by scaling transcripts per million. Meanwhile, relationships between DEGs and DAMs in typical HLB-affected pathways were analyzed.

## Materials and Methods

### Plant Materials and Inoculation

Seeds of Chongyi wild mandarin (*C. reticulata*) were sampled in Chongyi County, Jiangxi province, China. One-year-old seedlings were grafted with healthy (control) and *C*Las-infected buds from sweet orange trees. All the plants were maintained in the same greenhouse (at 25–28°C). The leaves were monitored biweekly by nested PCR to detect *C*Las. After 18 weeks, leaves, barks, and roots from *C*Las-positive and healthy seedlings were collected for paraffin sectioning, RNA-Seq, and ultra-high performance liquid tandem chromatography quadrupole time of flight mass spectrometry (UPLC-QTOFMS).

### Paraffin Sectioning

Tissues were fixed in formaldehyde-acetic acid-ethanol fixative, dehydrated in ethanol, and then embedded in paraffin. After being deparaffinized with xylene and stained with a fast green, the sliced sections were analyzed with a DM6 B (Leica, Wetzlar, Germany) microsystem combined with a DFC 7000T (Leica, Wetzlar, Germany) camera.

### Transcriptome Profiling and Data Processing

The RNA-Seq libraries were constructed, and high-throughput sequencing was carried out by Novogene (Beijing, China) using HiSeq 2000 (Illumina, San Diego, CA, United States). All sequencing adaptors, unknown nucleotides, and low-quality reads (quality score <20) were removed from the raw reads, and the resulting clean reads were used in subsequent analyses. HISAT2 (Kim et al., 2015) was performed to map the clean reads to the sweet orange (*Clonorchis sinensis*) genome (Xu et al., 2013). StringTie was used to estimate the abundance of transcripts in each sample (Pertea et al., 2016), and then the information of all the transcripts was assembled into a single gene transfer format (GTF) file using the “merge” option. Using the resulting GTF file as reference, StringTie was again used to obtain total gene expression information. Subsequently, the R subread package was applied to compute the number of uniquely mapping reads unambiguously attributed to each gene (Liao et al., 2019), which was used as input in DESeq2 (Love et al., 2014). A matrix of TPM per gene across all samples was generated to compare gene expression differences among the tissues.

### DEG Detection and Functional Enrichment

To check for similarities among these samples, a principal component analysis (PCA) in DESeq2 was performed based on per-gene unique reads counts. Genes with a total read number across all samples ≥18 were kept. Based on the same dataset, a clustering analysis was also performed using hclust in the “the stats” package in R (http://www.R-project.org) to detect clusters among all the 18 samples. Samples in unreasonable clusters were removed.

The DESeq2 package was used for DEGs detection between *C*Las-infected barks (B), leaves (L), and roots (R), and their corresponding controls (CY), namely, HLB_B vs. CY_B, HLB_L vs. CY_L, and HLB_R vs. CY_R. Before analysis, genes with several reads less than the total number of samples were filtered. Genes with a false discovery rate (FDR) <0.05 and log2 (Fold Change) [|log2(FoldChange)| >1] were considered as putative DEGs. Sequence similarity comparisons of the *C. sinensis* genome were performed with *Arabidopsis thaliana* using Blastx. Using *C. sinensis* as background, KOBAS 3.0 (Ai and Kong, 2018) was employed to annotate the Kyoto Encyclopedia of Genes and Genomes (KEGG) pathways of DEGs.

### Expression Normalization and Expression Pattern

Genes with zero TPM were filtered. The absolute gene expression, using normalized TPM value, was calculated according to Brawand et al. (2011). In detail, genes with a TPM value in the interquartile range (25–75%) of each sample were selected. Variance per gene across all samples was calculated to yield 1,000 least-varying genes and obtain median TPM values in each sample. Then, these median TPM values were used to derive the scaling factor of each sample, which was then utilized to normalize all the TPM values. The normalized TPM value matrix of all the genes was then used to estimate gene expression differences among all the tissues.

To investigate gene expression variations among the tissues, common DEGs identified in all three comparisons were filtered to estimate gene expression patterns among the tissues. Based on the normalized TPM values of these DEGs, the average TPM of each gene across biological replicates was presented as the expression level of this gene in the corresponding tissue, and the average was estimated by dividing the maximal value in all the tissues to generate relative expression profiles (Darbellay and Necsulea, 2020). Finally, the relative expression profiles were clustered with the K-means algorithm in R to investigate gene expression patterns among the barks, leaves, and roots.

### Metabolome Profiling and Data Processing

The UPLC-QTOFMS analysis was carried out by Biotree (Shanghai, China). Quality control (QC) samples were prepared by mixing the same volume from all the samples. The UPLC-QTOFMS analysis was performed on 1290 Infinity LC System (Agilent, Sta. Clara, CA, United States) coupled to an Agilent 6538 Accurate-Mass Quadrupole Time-of-Flight (Q-TOF) (Agilent, Sta. Clara, CA, United States) mass spectrometer. The metabolomic data were collected in a centroid mode and the mass range was set at 50–1,100 m/z using an extended dynamic range.

The raw metabolomics data were converted into a normal file (.mzXML) using MSConvert (ProteoWizard 3.0). MZmine 2.1 (Pluskal et al., 2010) was applied for identifying features, deisotopes, feature alignments, and gap-filling to avoid missing some features in the first alignment algorithm. After removing the complexes, all adducts were searched against an internal retention time metabolite library. Data on both positive and negative ions were subjected to statistical analysis. Tables with metabolite peaks (mz/rt) of all the three tissues under both healthy and symptomatic conditions were formatted as comma-separated values (.csv). The resulting three-dimensional data of the peak number, sample name, and normalized peak area were fed to the SIMCA14.1 software package (V14.1; MKS Data Analytics Solutions, Umea, Sweden) for orthogonal projections to latent structures discriminant analysis (PLS-DA).

The DAMs were selected based on a statistically significant threshold of variable influence on projection (VIP) values acquired from the PLS-DA (VIP ≥1 and *p* ≤ 0.05). MetaboAnalyst was employed for KEGG enrichment analysis (Xia and Wishart, 2016). The DAVID Functional Annotation tool was applied to investigate the interactions of DEGs and DAMs in pathways (Huang et al., 2009).

### Putative Marker Gene Identification for Symptomatic Tissues

Gene expression specificity across tissues was estimated using the equation: $\text{tau}=\sum_{i=1}^{n}\left(1-ri\right)/(n-1)$, where *r*_*i*_represents the ratio between the expression level (normed TPM value) of a gene in sample *i* and the maximal expression level across samples (Liao et al., 2006). The average tau value of all the biological replicates was calculated and used as the specific expression level for a given tissue. The tau value ranged from 0 to 1. A higher tau value indicated greater variation in expressional level across tissues, namely, higher tissue specificity.

To identify the putative markers for HLB infection, genes with expression levels (TPM) ≥2 in at least one sample of each tissue were selected. The putative marker genes should have a high expression specificity index (tau ≥ 0.85) in a specific healthy tissue and maximal expression in the corresponding symptomatic tissue (Liao et al., 2006). Due to the shortage of Chongyi samples, some putative marker genes were verified by qRT-PCR with the *C*Las-infected and healthy leaves from *C. sinensis*.

## Results

### Confirmation of HLB and Anatomical Aberrations in *C*Las-Infected Tissues

**Figure 1:**
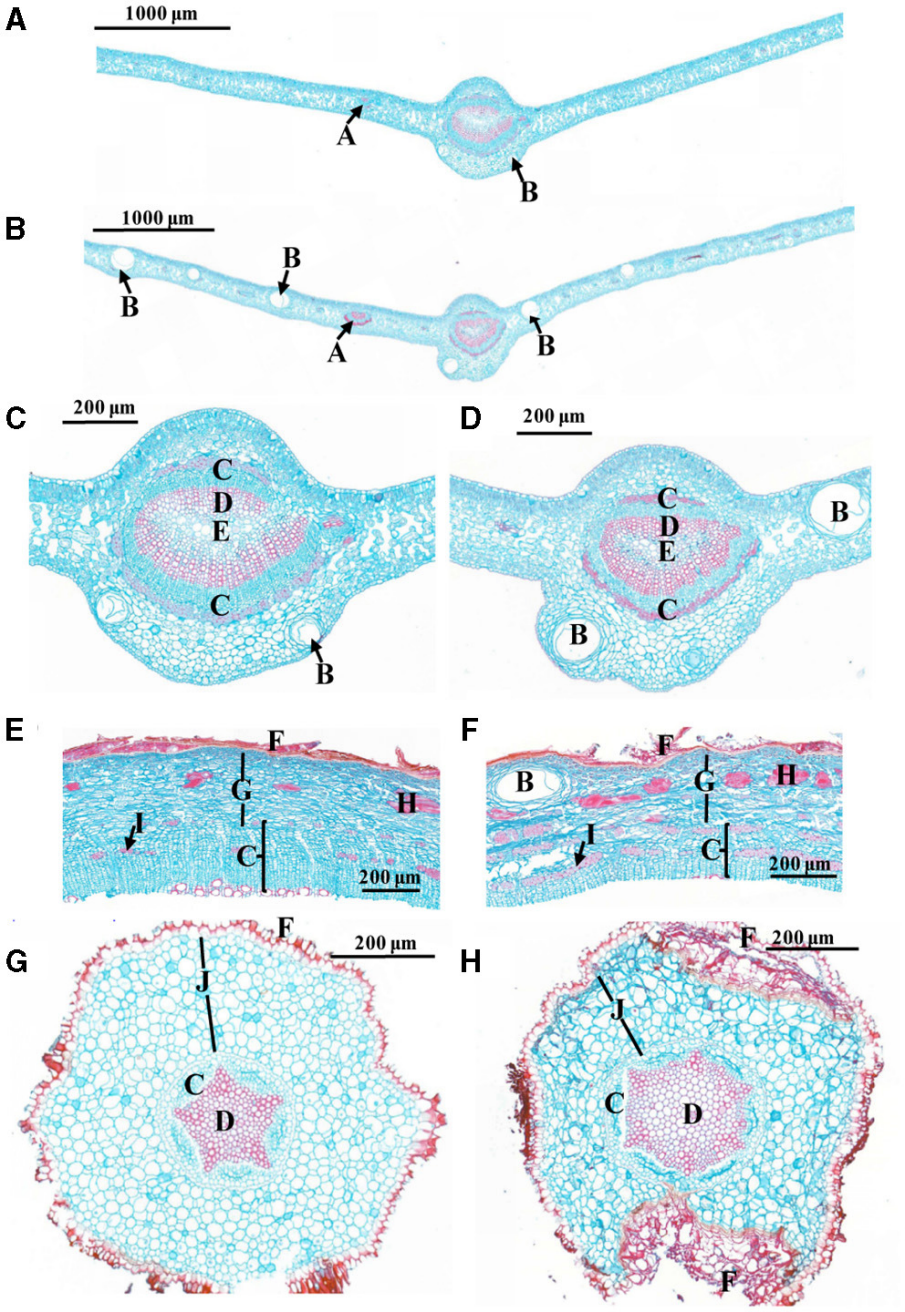
Comparisons of cross-sections between healthy **(A,C,E,G)** and C. L. asiaticus (*C*Las)-infected **(B,D,F,H)** tissues. **(A–D)** Leaves; **(C,D)** are the close-ups of midvein from **(A,B)**; **(E,F)** barks; **(G,H)** roots. A: vascular bundle of lateral veinlet; B: air cavity; C: phloem; D: xylem; E: pith; F: epidermis; G: parenchyma cell; H: sclereid; I: phloem fiber; J: cortex.

After 18 weeks of grafting, all the *C*Las-inoculated Chongyi seedlings were positive by Nested PCR amplification (Supplementary Figure 1). Subsequently, anatomical differences between the *C*Las-positive and healthy tissues were compared. More enlarged vascular bundles of lateral veinlets and air cavities were observed in the *C*Las-infected leaves (Figures 1A,B). Cells of phloem, xylem, parenchyma, and cortex arranged irregularly after *C*Las infection (Figure 1). Particularly, more sclereids and phloem fibers accumulated in the *C*Las-infected barks (Figures 1E,F). In the *C*Las-infected roots, the cortical tissue collapsed and separated from the epidermis (Figures 1G,H). Approximately 1 year after inoculation, the remaining *C*Las-positive Chongyi seedlings died out. Therefore, Chongyi was considered to be susceptible to HLB.

### DEGs and DAMs Induced by *C*Las Inoculation

**Figure 2:**
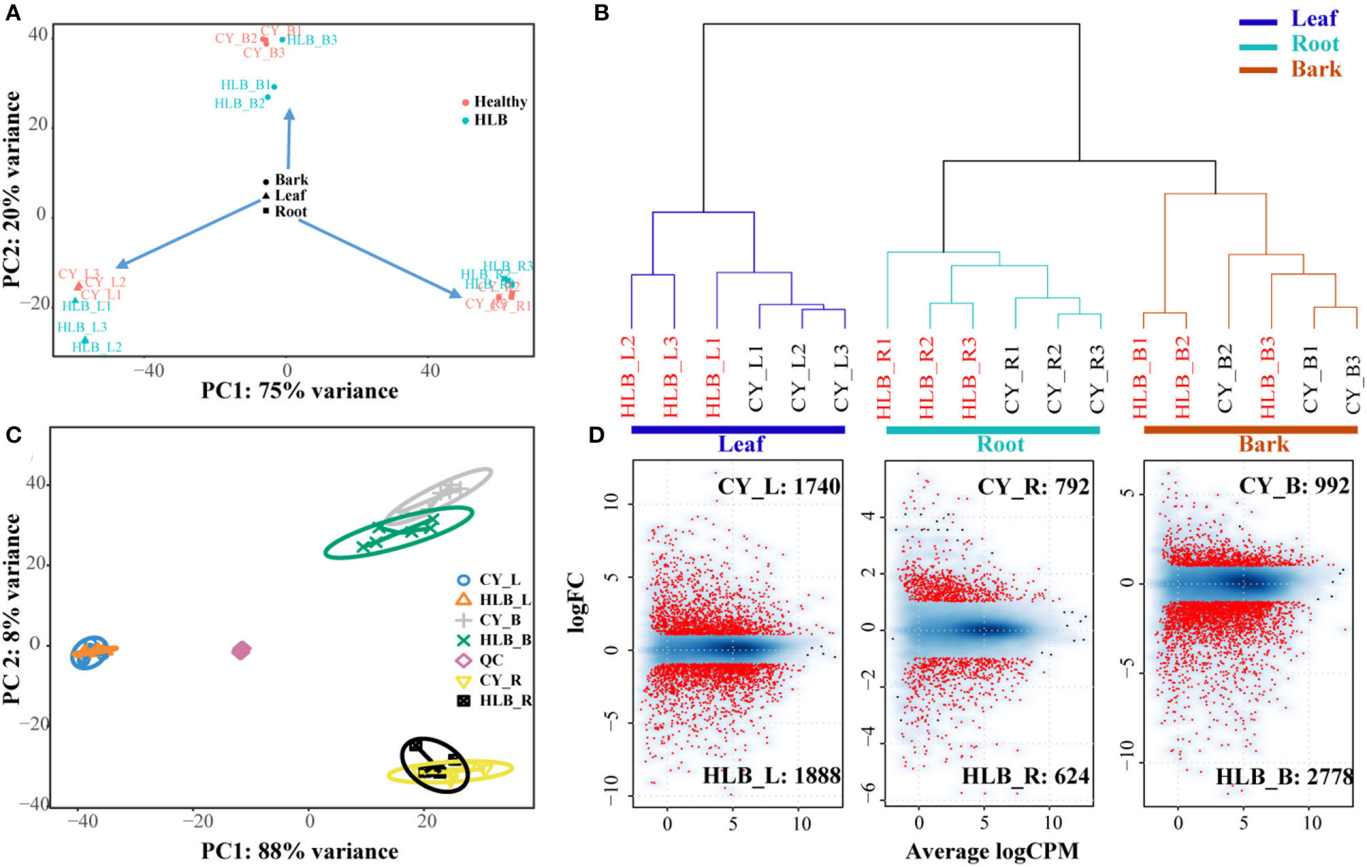
Cluster analysis of all the differentially expressed genes (DEGs) and detected metabolites between *C*Las-infected and healthy tissues. **(A)** Principal component analysis (PCA) analysis of all the samples based on all the expressed genes; **(B)** Hclust analysis of all the samples based on all the expressed genes; **(C)** sPLS-DA analysis of all the samples based on all of the metabolites; **(D)** analysis of DEG detection: a smooth scatter was used to convert the number of points in each plot coordinate into a vector of colors representing the local point density.

Around 83.34–86.06% of the clean reads could be mapped to the *C. sinensis* genome (Supplementary Table 1). Genes with a total read number over 18 were used for PCA analysis. The PCA results indicated that principal component 1 (PC1) could explain about 75% variances, and PC2 could explain about 20% variances (Figure 2A). All of the samples could be divided into three main clusters in PC1 according to the tissue type, and PC2 separated these samples depending on whether a sample was healthy or *C*Las-infected. An Hclust analysis with all of the expressed genes yielded similar results, i.e., there were three main clusters (leaves, roots, and barks), and the root samples were clustered with the bark samples (Figure 2B). However, the two results both indicated that HLB_L1 was clustered with the healthy leaves and that HLB_B3 was clustered with the healthy barks. Therefore, HLB_L1 and HLB_B3 were excluded from the subsequent analysis.

For the metabolites, there were 2,541 and 2,003 peaks identified in the negative and positive ion modes. Of these peaks, 130 and 193 could be mapped as known metabolites, which included amino acids, organic acids, sugars, polyamines, nitrogenous compounds, and polyphenols. Three tissue-specific groups were clustered by the sPLS-DA, whereas PC1and PC2 explained 88 and 8%variances among all of the metabolomic samples, respectively (Figure 2C). From the heatmap of all the detected metabolites (Supplementary Figure 2), more noticeable differences were observed among the *C*Las-infected and healthy tissues.

There were 3,628 DEGs in the leaves, 3,770 in the barks, and 1,716 in the roots, among which 1,888 in HLB_L, 2,778 in HLB_B, and 624 in HLB_R were upregulated, and 1,740 in CY_L, 992 in CY_B, and 792 in CY_R were upregulated (Figure 2D). Meanwhile, 3,240 DEGs in the barks (Supplementary Table 2), 3,038 in the leaves (Supplementary Table 3), and 1,216 in the roots (Supplementary Table 4) had citrus gene IDs. For the KEGG pathway enrichment analysis, 24,376 coding sequences of the longest transcript in *C. sinensis* were extracted to represent the corresponding genes. DEGs with a coding gene sequence were used as input sequences in KOBAS.

Metabolite intensities of the healthy and *C*Las-positive samples among the three tissues were compared. Subsequently, 62 DAMs in the barks (Table 1), 71 in the leaves (Table 2), and 50 in the roots (Table 3) could be matched to a corresponding KEGG compound ID. The majority of DAMs in the *C*Las-infected barks and leaves were increased, but half in the *C*Las-infected roots were decreased, suggesting that HLB affected roots more greatly at the metabolic level.

**Table 1:** Differentially accumulated metabolites (DAMs) between C. L. asiaticus (*C*Las)-infected and healthy barks.

**No**.	**Metabolites**	**VIP^**a**^**	***P*-value^**b**^**	**Log2FoldChange^**c**^**	**KEGG compound id**
1	Palmitic acid	1.52	7.73E-04	3.37	C00249
2	Sinapyl alcohol	1.79	1.79E-03	2.19	C02325
3	Thiamine monophosphate	1.30	1.29E-03	1.93	C01081
4	S-Methyl-5'-thioadenosine	1.39	1.62E-04	1.89	C00170
5	Myristic acid	1.43	1.14E-03	1.69	C06424
6	L-Carnitine	1.55	6.25E-10	1.62	C00318
7	Acetylcarnitine	1.50	4.86E-04	1.55	C02571
8	Salicyluric acid	1.69	2.15E-03	1.53	C07588
9	L-Glutamate	1.71	4.26E-03	1.53	C00025
10	4-Pyridoxic acid	1.43	4.01E-06	1.50	C00847
11	Folinic acid	1.50	1.76E-06	1.32	C03479
12	Mevalonic acid	1.36	4.01E-04	1.23	C00418
13	Indole-3-carboxylic acid	1.50	2.09E-04	1.17	C19837
14	L-Glutamine	1.52	1.54E-02	1.11	C00064
15	3,4-Dihydroxyhydrocinnamic acid	1.39	1.39E-04	1.03	C10447
16	N-Acetyl-L-aspartic acid	1.13	8.72E-03	0.97	C01042
17	L-Arginine	1.48	3.32E-04	0.94	C00062
18	Sucrose	1.53	4.45E-08	0.90	C00089
19	N-Acetylmannosamine	1.13	3.44E-03	0.88	C00645
20	Acetyl-DL-Leucine	1.52	5.83E-05	0.88	C02710
21	D-(+)-Galactose	1.49	3.92E-06	0.86	C00984
22	Vanillic acid	1.53	5.61E-03	0.85	C06672
23	Gentisic acid	1.21	7.74E-04	0.85	C00628
24	D-Mannose	1.49	7.85E-06	0.81	C00159
25	Azelaic acid	1.18	8.95E-03	0.80	C08261
26	Tetracosanoic acid	1.35	5.42E-04	0.80	C08320
27	Homovanillic acid	1.63	6.66E-03	0.75	C05582
28	Prunasin	1.41	1.65E-04	0.74	C00844
29	L-Asparagine	1.25	2.97E-03	0.73	C00152
30	5,10-methylene-THF	1.36	2.84E-02	0.70	C00143
31	L-Serine	1.45	7.90E-06	0.69	C00065
32	Vanillin	1.31	3.28E-04	0.67	C00755
33	Uric acid	1.32	4.50E-03	0.67	C00366
34	Stearic acid	1.41	5.11E-05	0.63	C01530
35	2-Isopropylmalic acid	1.07	1.29E-02	0.63	C02504
36	N-Acetyl-L-phenylalanine	1.20	4.06E-02	0.62	C03519
37	Palmitaldehyde	1.45	4.15E-03	0.60	C00517
38	Phenol	1.21	5.18E-03	0.59	C00146
39	Methylmalonic acid	1.36	1.39E-02	0.58	C02170
40	Rutin	1.44	1.93E-02	0.56	C05625
41	Dihydrouracil	1.16	1.10E-02	0.54	C00429
42	Synephrine	1.15	5.45E-03	0.52	C04548
43	7,8-Dihydrofolate	1.08	1.15E-02	0.46	C00415
44	D-galacturonic acid	1.14	2.52E-02	0.44	C08348
45	N-Acetyl-D-galactosamine	1.04	4.98E-02	0.43	C01074
46	Oleoyl-CoA	1.31	1.35E-02	0.35	C00510
47	Adenosine	1.21	4.52E-03	0.30	C00212
48	Adenine	1.06	1.86E-02	0.27	C00147
49	1-Deoxy-D-xylulose 5-phosphate	1.09	4.39E-02	−0.26	C11437
50	Homocitrate	1.14	7.13E-03	−0.30	C01251
51	Xylitol	1.18	5.46E-03	−0.38	C00379
52	Alpha-ketoisovaleric acid	1.07	1.32E-02	−0.39	C00141
53	Phytosphingosine	1.06	1.28E-02	−0.64	C12144
54	Linoleic acid	1.34	3.58E-04	−0.66	C01595
55	Maleic acid	1.27	3.20E-03	−0.74	C01384
56	Dihydroxyacetone	1.17	1.07E-02	−0.75	C00184
57	5'-Deoxyadenosine	1.30	1.21E-03	−0.84	C05198
58	Phosphorylcholine	1.34	3.57E-04	−0.92	C00588
59	D-Tagatose	1.25	2.22E-02	−0.96	C00795
60	L-Iditol	1.22	1.20E-02	−1.19	C01507
61	PA [16:0/18:2(9Z,12Z)]	1.39	2.64E-04	−1.32	C00416
62	D-Threitol	1.51	1.06E-04	−2.59	C16884

a *VIP: the importance of the variable projection of substance in the orthogonal projections to latent structures discriminant analysis (OPLS-DA) model*.

b *P-value: the P-value of substance in the t-test of the group*.

c *Log2FoldChange: the logarithm of the fold change of DAMs between CLas-infected and healthy roots to the base 2*.

**Table 2:** DAMs between *C*Las-infected and healthy leaves.

**No**.	**Metabolites**	**VIP^**a**^**	***P*-value^**b**^**	**Log2FoldChange^**c**^**	**KEGG compound id**
1	Rutin	1.38	2.12E-02	2.73	C05625
2	Xanthurenic acid	1.04	2.43E-05	2.23	C02470
3	D-Mannitol	1.55	2.52E-07	2.10	C00392
4	Scopoletin	1.28	2.04E-02	1.70	C01752
5	4-Hydroxybenzoate	1.51	1.14E-06	1.70	C00156
6	Dopamine	1.54	5.34E-08	1.68	C03758
7	Vanillin	1.54	4.74E-05	1.59	C00755
8	4-Hydroxyphenylpyruvate	1.34	1.42E-02	1.40	C01179
9	D-Proline	1.43	2.44E-06	1.37	C00763
10	Glycitein	1.34	3.65E-03	1.19	C14536
11	L-Proline	1.50	1.88E-06	1.17	C00148
12	Phloretin	1.53	8.91E-06	1.13	C00774
13	Gentisic acid	1.48	1.76E-05	1.09	C00628
14	Maleic acid	1.47	6.97E-06	1.07	C01384
15	Vanillic acid	1.34	3.79E-03	1.06	C06672
16	Apigenin	1.30	1.87E-03	0.96	C01477
17	L-Glutamine	1.39	9.50E-05	0.89	C00064
18	LysoPC(16:0)	1.41	8.80E-05	0.85	C04230
19	Mevalonic acid	1.42	4.74E-05	0.83	C00418
20	5,10-methylene-THF	1.32	5.97E-04	0.82	C00143
21	L-Carnitine	1.42	2.01E-05	0.82	C00318
22	Guanosine	1.35	7.43E-04	0.79	C00387
23	N-Acetylmannosamine	1.12	1.42E-02	0.72	C00645
24	Phenol	1.26	2.70E-03	0.70	C00146
25	5'-Deoxyadenosine	1.01	2.30E-02	0.66	C05198
26	D-(+)-Galactose	1.33	2.36E-04	0.65	C00984
27	Gamma-Butyrolactone	1.17	4.82E-03	0.64	C01770
28	Homocitrate	1.28	1.31E-03	0.61	C01251
29	Capecitabine	1.18	7.14E-03	0.60	C12650
30	Synephrine	1.28	1.08E-03	0.57	C04548
31	Oleoyl-CoA	1.32	8.79E-04	0.53	C00510
32	Isomaltose	1.25	2.51E-03	0.51	C00252
33	D-Maltose	1.21	1.60E-02	0.49	C00208
34	L-Pyroglutamic acid	1.08	1.12E-02	0.49	C01879
35	Thiamine	1.41	1.26E-04	0.47	C00378
36	D-Mannose	1.03	2.39E-02	0.41	C00159
37	1,2,3-Trihydroxybenzene	1.14	8.66E-03	0.40	C01108
38	Sucrose	1.14	7.63E-03	0.37	C00089
39	3-Methylindole	1.28	2.13E-03	0.35	C08313
40	2-Ethoxyethanol	1.33	1.20E-03	0.30	C14687
41	Adenosine	1.06	1.96E-02	0.28	C00212
42	O-Phosphoethanolamine	1.19	7.30E-03	0.21	C00346
43	cis-9-Palmitoleic acid	1.21	2.47E-03	−0.28	C08362
44	Indoleacetic acid	1.09	1.32E-02	−0.34	C00954
45	Aminocaproic acid	1.16	7.61E-03	−0.43	C02378
46	L-Malic acid	1.03	1.88E-02	−0.47	C00149
47	beta-Hydroxybutyrate	1.24	1.08E-02	−0.48	C01089
48	2'-Deoxy-D-ribose	1.22	5.57E-03	−0.49	C01801
49	4-Hydroxycinnamic acid	1.25	2.17E-03	−0.49	C00811
50	PA(16:0/18:1(9Z))	1.21	3.41E-03	−0.51	C00416
51	Curcumin	1.55	5.09E-07	−0.59	C10443
52	1-Deoxy-D-xylulose 5-phosphate	1.24	2.85E-03	−0.59	C11437
53	Stearic acid	1.40	8.21E-05	−0.59	C01530
54	N-Acetyl-L-glutamate	1.07	1.13E-02	−0.59	C00624
55	trans-Vaccenic acid	1.21	5.53E-03	−0.65	C08367
56	Gamma-Glutamylcysteine	1.32	9.07E-04	−0.66	C00669
57	Phenylacetylglycine	1.02	3.83E-02	−0.66	C05598
58	L-Citrulline	1.18	9.17E-03	−0.73	C00327
59	Nicotinamide	1.05	1.26E-02	−0.74	C00153
60	4-Pyridoxic acid	1.22	3.15E-03	−0.76	C00847
61	Cyclohexylamine	1.29	1.10E-02	−0.80	C00571
62	Glycerophosphocholine	1.09	2.69E-02	−0.80	C00670
63	Pantothenate	1.33	2.77E-04	−0.84	C00864
64	L-Pipecolic acid	1.22	3.41E-02	−0.94	C00408
65	Prunasin	1.41	1.32E-03	−1.24	C00844
66	Isorhamnetin	1.42	4.78E-03	−1.29	C10084
67	D-Tagatose	1.43	1.38E-05	−1.35	C00795
68	17-beta-Estradiol-3-glucuronide	1.47	4.07E-04	−1.38	C05503
69	Dihydroxyacetone	1.44	1.05E-05	−1.60	C00184
70	Inosine	1.52	3.28E-07	−1.67	C00294
71	Raffinose	1.53	1.14E-04	−1.73	C00492

a *VIP: importance of the variable projection of substance in the OPLS-DA model*.

b *P-value: the P value of substance in the t-test of the group*.

c *Log2FoldChange: logarithm of the fold change of DAMs between CLas-infected and healthy leaves to base 2*.

**Table 3:** DAMs between *C*Las-infected and healthy roots.

**No**.	**Metabolites**	**VIP^**a**^**	***P*-value^**b**^**	**Log2FoldChange^**c**^**	**KEGG compound id**
1	Sinapyl alcohol	1.79	1.79E-03	2.19	C02325
2	Salicyluric acid	1.69	2.15E-03	1.53	C07588
3	L-Glutamate	1.71	4.26E-03	1.53	C00025
4	L-Glutamine	1.52	1.54E-02	1.11	C00064
5	5,10-methylene-THF	1.72	7.44E-03	1.02	C00143
6	Palmitic acid	1.34	1.58E-02	0.97	C00249
7	3,4-Dihydroxyhydrocinnamic acid	1.12	3.85E-02	0.93	C10447
8	Thiamine	2.01	8.61E-05	0.88	C00378
9	Vanillic acid	1.53	5.61E-03	0.85	C06672
10	Azelaic acid	1.40	2.97E-02	0.80	C08261
11	Homovanillic acid	1.63	6.66E-03	0.75	C05582
12	2-Isopropylmalic acid	1.38	6.79E-03	0.62	C02504
13	N-Acetyl-L-phenylalanine	1.20	4.06E-02	0.62	C03519
14	Palmitaldehyde	1.45	4.15E-03	0.60	C00517
15	Methylmalonic acid	1.36	1.39E-02	0.58	C02170
16	Maleic acid	1.50	3.62E-03	0.57	C01384
17	Rutin	1.44	1.93E-02	0.56	C05625
18	D-galacturonic acid	1.14	2.52E-02	0.44	C08348
19	Stearic acid	1.34	9.96E-03	0.43	C01530
20	N-Acetyl-D-galactosamine	1.04	4.98E-02	0.43	C01074
21	Xylitol	1.45	2.70E-03	0.42	C00379
22	5'-Deoxyadenosine	1.26	1.18E-02	0.40	C05198
23	Uric acid	1.13	4.09E-02	0.40	C00366
24	Alpha-ketoisovaleric acid	1.16	1.75E-02	0.36	C00141
25	Oleoyl-CoA	1.31	1.35E-02	0.35	C00510
26	1-Deoxy-D-xylulose 5-phosphate	1.09	4.39E-02	−0.26	C11437
27	DL-3-Phenyllactic acid	1.49	1.91E-02	−0.40	C01479
28	Curcumin	1.56	1.65E-02	−0.43	C10443
29	Folinic acid	1.73	1.06E-03	−0.49	C03479
30	N-Acetylputrescine	1.78	6.30E-04	−0.57	C02714
31	L-Palmitoylcarnitine	1.56	1.05E-02	−0.58	C02990
32	Adenosine	1.57	1.42E-02	−0.59	C00212
33	D-Maltose	1.49	1.59E-02	−0.60	C00208
34	D-Mannitol	1.95	2.93E-04	−0.60	C00392
35	Isovaleric acid	2.07	4.24E-06	−0.63	C08262
36	Guanosine	1.75	3.57E-03	−0.65	C00387
37	Scopoletin	1.86	5.43E-04	−0.67	C01752
38	Capecitabine	1.73	2.77E-03	−0.69	C12650
39	1,2,3-Trihydroxybenzene	1.58	9.37E-03	−0.69	C01108
40	Glycerophosphocholine	1.81	1.38E-03	−0.73	C00670
41	Folate	1.91	1.78E-04	−0.73	C00504
42	Diethanolamine	1.51	6.28E-03	−0.76	C06772
43	Phenylacetylglycine	1.82	2.77E-03	−0.77	C05598
44	D-Mannose	1.56	1.03E-02	−0.80	C00159
45	Isomaltose	1.59	8.97E-03	−0.80	C00252
46	Glycitein	1.82	4.46E-03	−0.99	C14536
47	Maltotriose	1.90	1.13E-03	−1.09	C01835
48	4-Hydroxycinnamic acid	1.81	2.65E-04	−1.18	C00811
49	Pyrocatechol	1.99	4.15E-03	−1.47	C00090
50	2'-Deoxy-D-ribose	1.97	6.19E-03	−1.67	C01801

a *VIP: the importance of the variable projection of substance in the OPLS-DA model*.

b *P-value: the P-value of substance in the t-test of the group*.

c *Log2FoldChange: logarithm of the fold change of DAMs between CLas-infected and healthy roots to base 2*.

**Figure 3:**
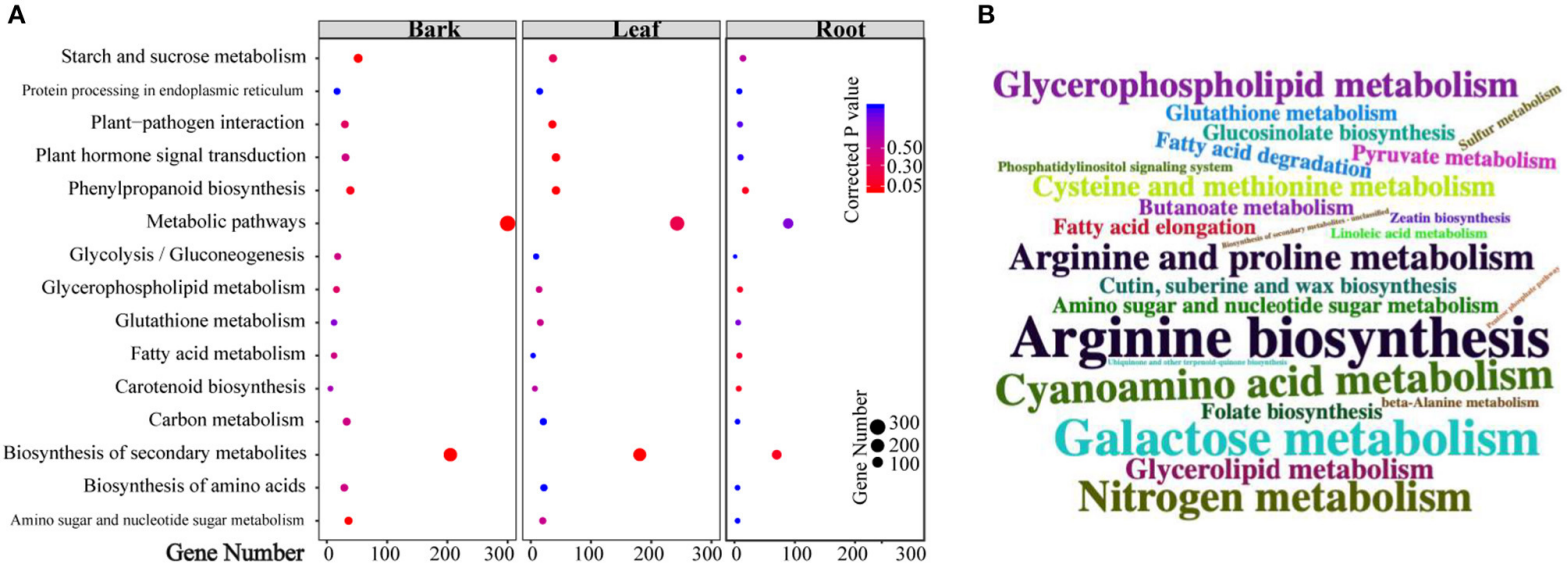
Kyoto Encyclopedia of Genes and Genomes (KEGG) pathway enrichment analysis of differentially expressed genes (DEGs) and differentially accumulated metabolites (DAMs). **(A)** Pathways that enriched top10 gene numbers in each comparison between *C*Las-infected and healthy tissues; **(B)** pathways that enriched detected metabolites: larger size means more metabolites were detected in this pathway.

Based on the number of DEGs enriched in the KEGG pathways (Supplementary Tables 5–7), the top 10 pathways in each tissue are shown in Figure 3A. Moreover, “metabolic pathways” had the highest number of DEGs in all the three tissues (barks: 300 DEGs, corrected *p*-value (CP) = 6.10E-5; leaves: 243 DEGs, CP = 0.232; roots: 80 DEGs, CP = 0.832), followed by “biosynthesis of secondary metabolites” (barks: 205 DEGs, CP = 4.23E-07; leaves: 181 DEGs, CP = 0.0001). Among all the significantly enriched pathways, “starch and sucrose metabolism,” “glycosaminoglycan degradation,” “plant-pathogen interaction,” “nitrogenmetabolism,” “carotenoid biosynthesis,” and “plant hormone signal transduction” were shared in all three tissues.

Similarly, more DAMs were enriched in “metabolic pathways” and “biosynthesis of secondary metabolites” in the three tissues (Figure 3B; Supplementary Tables 8–10). In addition, 56 pathways in the barks (Supplementary Table 11), 54 in the leaves (Supplementary Table 12), and 45 in the roots (Supplementary Table 13) displayed significant changes in both gene expression and metabolite content, suggesting that the results of transcriptomics and metabolomics were consistent with each other.

### Common DEGs in the Three Tissues Exhibited Eight Expression Patterns

**Figure 4:**
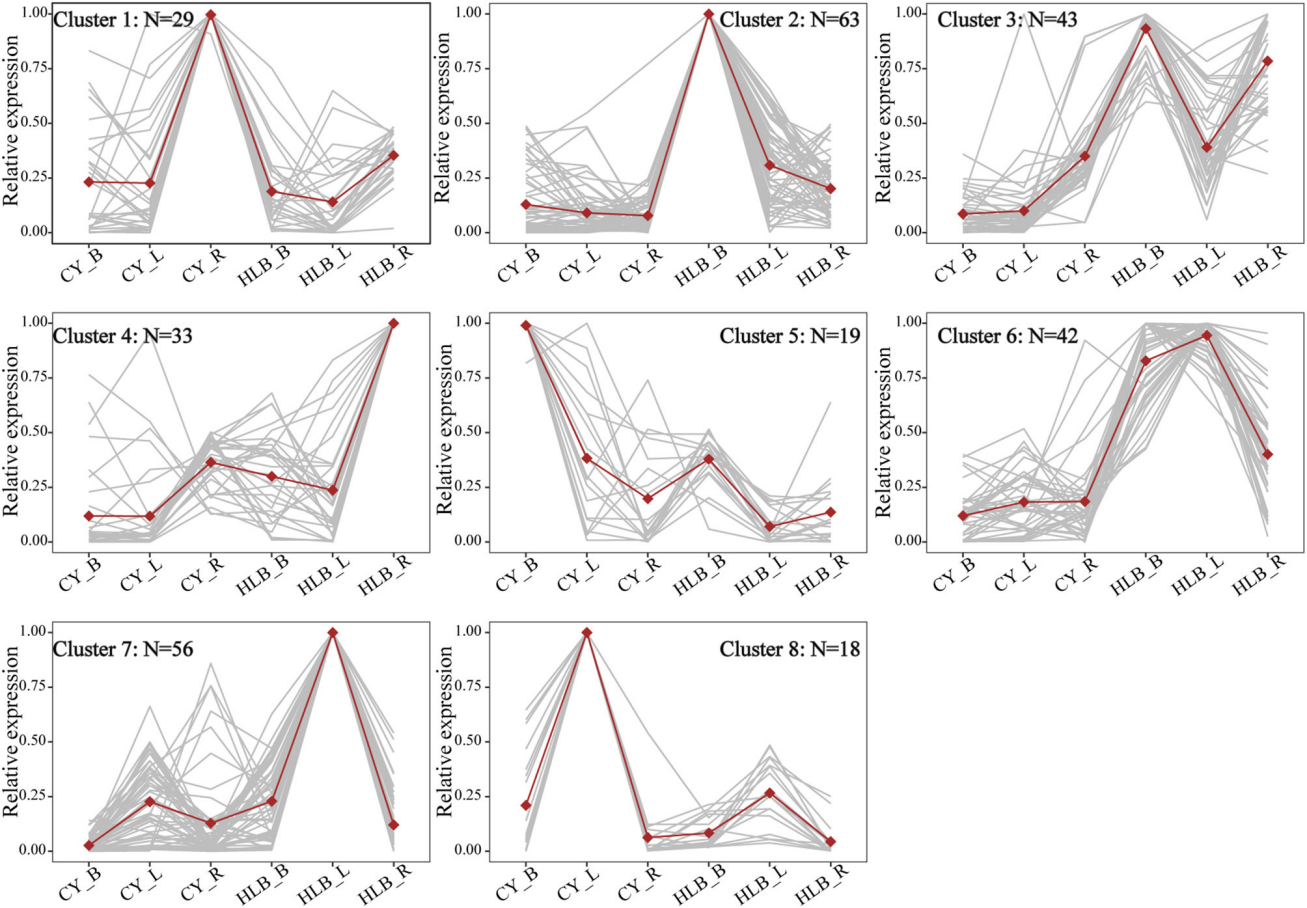
Expression patterns of common DEGs among all tissues. CY_B, CY_L, and CY_R represent healthy barks, leaves, and roots, respectively. HLB_B, HLB_Y, and HLB_R represent *C*Las-infected barks, leaves, and roots, respectively. Normalized TPM values were averaged across replicates for each tissue. Dots represent the average profiles of genes belonging to each cluster. Gray lines represent the profiles of individual genes from a cluster.

To compare expression variation among the three tissues, the expression patterns of common DEGs were analyzed. There were 304 common DEGs shared by the three tissues, while 1,884, 1,930, and 6,30 were specific to the leaves, barks, and roots, respectively (Supplementary Figure 3a). The clustering result from these 304 DEGs revealed that there was an increase in the sum of squares when the number of groups (K) was reduced from nine to eight (Supplementary Figure 3b). Thus, it might be proper to divide the 304 common DEGs into eight different expression patterns. As shown in Figure 4, DEGs in clusters 2 (*N* = 63), 3 (*N* = 43), 4 (*N* = 33), 6 (*N* = 4 2), and 7 (*N* = 56) were upregulated in the *C*Las-infected tissues, but the DEGs in clusters 1 (*N* = 29), 5 (*N* = 19), and 8 (*N* = 18) were downregulated.

Especially, six common DEGs of the three tissues involved in starch and sucrose metabolism are shown in Supplementary Figure 4, namely, *pectinesterase* (*PME1*), probable *trehalose-phosphate phosphatase D* (*TPPD*), *UDP-glucuronate 4-epimerase 1* (*GAE1*), probable *pectinesterase/pectinesterase inhibitor 44* (*PME44*), *beta-glucosidase 40* (*BGLU40*), and *trehalose 6-phosphate phosphatase* (*TPPA*). All of them were upregulated in HLB_L/B/R, except for *BGLU40*, which was downregulated in HLB_R. Therefore, significant changes among the pathways related to carbohydrates occurred at the transcriptional level.

### *C*Las-Infected Tissues Had Higher Transcripts Per Kilobase Per Million (TPM) Values and More Genes That Reached the Maximal Expression (GME)

**Figure 5:**
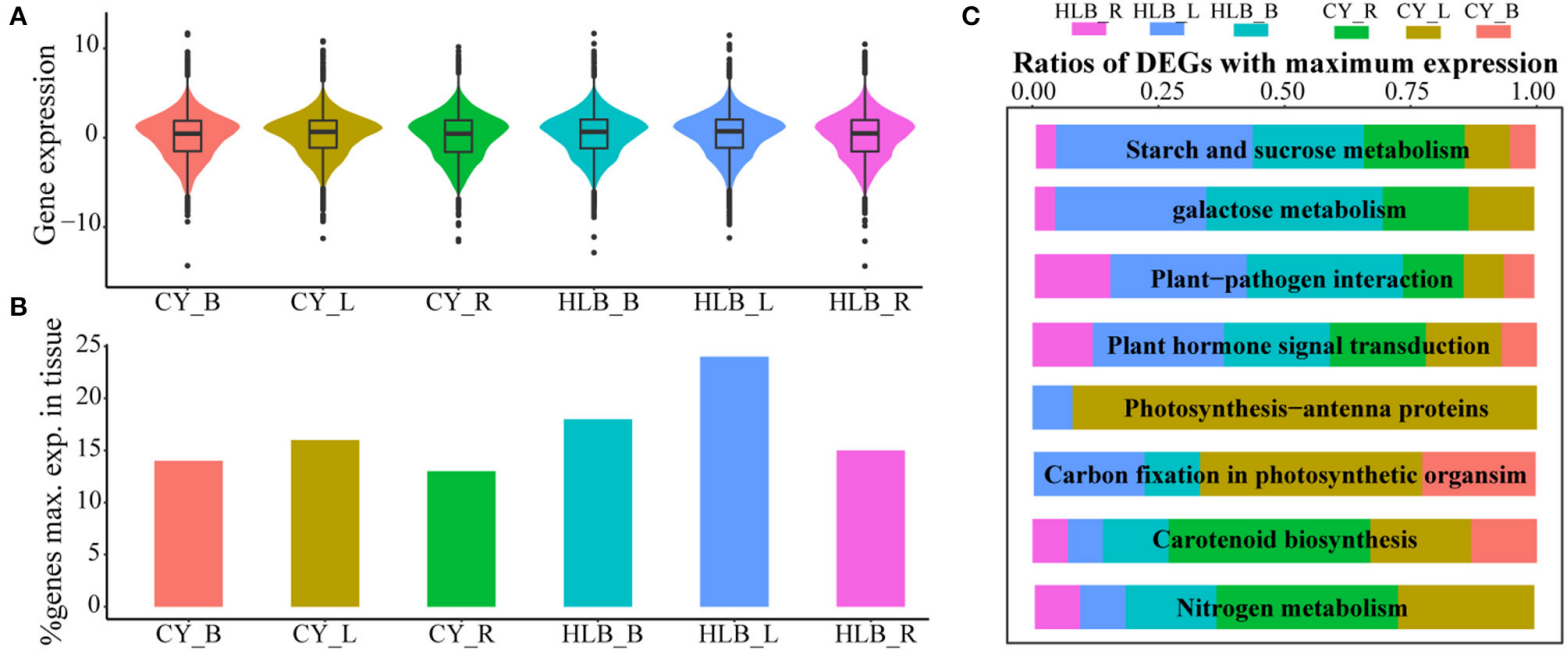
Relative gene expressions among all tissues. **(A)** Gene expression distributions among all tissues; **(B)** comparison of the expressed gene number with maximal expression among the three tissues. Genes of each tissue with maximal expression are computed based on average expression values across biological replicates; **(C)** relative ratios with maximal gene expression among eight typical HLB-affected pathways in all the tissues.

Expression levels of a total of 19,558 genes were normalized by scaling their TPM values, and the average of log2(normed TPM) of gene expression in biological replicates was represented as the expression level of this gene in the corresponding tissue. Median TPM values in the *C*Las-infected leaves, barks, and roots were 0.722, 0.657, and 0.485, respectively, and those in the corresponding control tissues were 0.661, 0.467, and 0.468. However, the TPM distribution of all the expressed genes was nearly the same among all the tissue samples (Figure 5A). As for the percentage of GMEs in each tissue, 24, 18, and 15% of the genes reached their maximal expressions in the *C*Las-infected leaves, barks, and roots, respectively, which were higher than those in the controls (Figure 5B). Higher TPM values and more GMEs in the *C*Las-infected tissues suggested that HLB infection might cause an overall increase in transcript accumulation.

However, GMEs in eight individual pathways varied (Figure 5C). In the pathways related to “starch and sucrose metabolism,” “galactose metabolism,” “plant-pathogen interaction,” and “plant hormone signal transduction,” the *C*Las-infected tissues had more genes that reached their maximal expression levels than the healthy tissues, suggesting that HLB significantly affected these pathways, especially in the *C*Las-infected leaves and barks (Figure 5C). Nevertheless, “carbon fixation in photosynthetic organism,” “photosynthesis-antenna protein,” “carotenoid biosynthesis” and “nitrogen metabolism” had more GMEs in the healthy tissues than in the *C*Las-infected tissues, indicating that HLB suppressed related physiological processes, particularly photosynthesis in leaves and carotenoid and nitrogen metabolism in roots (Figure 5C).

### DEGs Related to Early Signal Perception and Transduction

**Figure 6:**
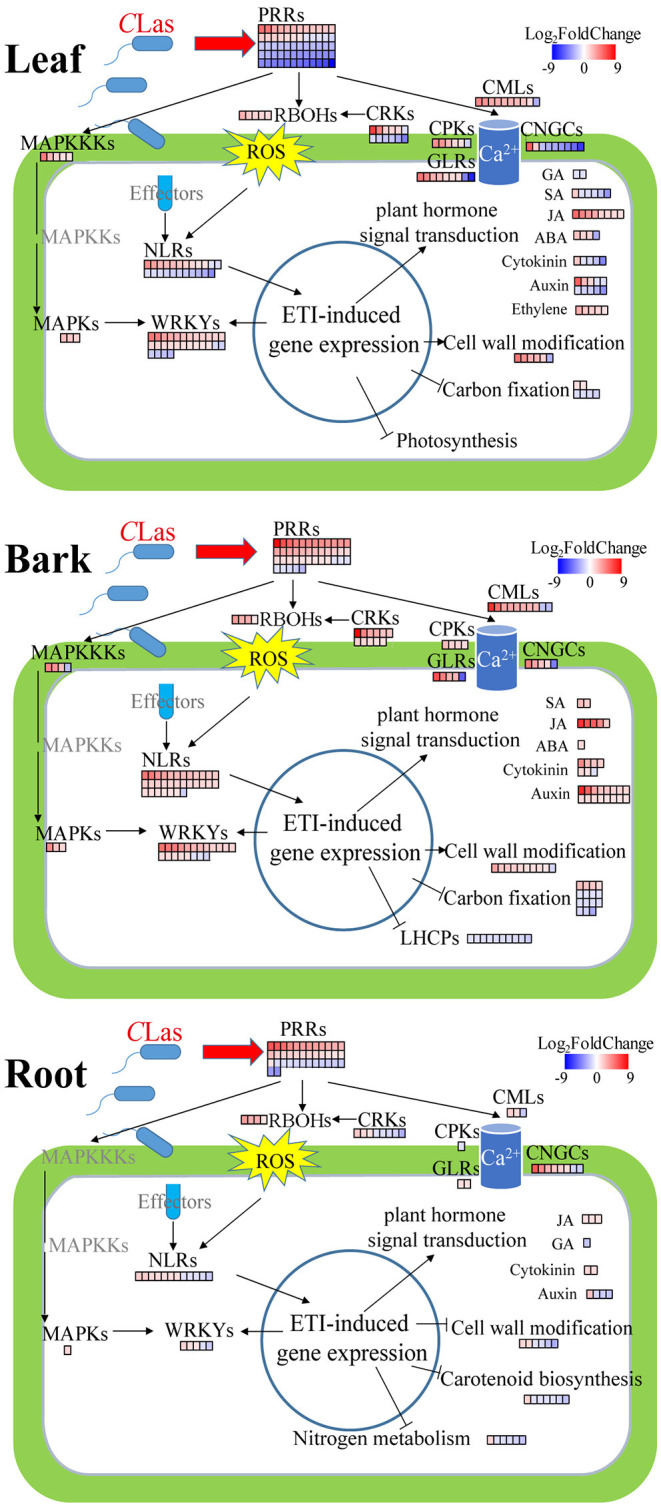
Summary of defense-related DEGs in *C*Las-infected leaves, barks, and roots. Red, upregulation; blue, downregulation. PRRs, pattern recognition receptors; RBOHs, plant respiratory burst oxidase homologs; ROS, reactive oxygen species; MAPKKKs, mitogen-activated protein kinase kinase kinases; MAPKs, mitogen-activated protein kinases; CMLs, calmodulin-like proteins; CPKs, calcium-dependent protein kinases; CNGCs, cyclic nucleotide-gated channels; CRKs, cysteine-rich receptor-like kinases; GLRs, glutamate-like receptors; NLRs, nucleotide-binding–leucine-rich-repeat receptors; ETI, effector-triggered immunity. MAPKKKs, MAPKs, CPKs, CRKs, and GLRs are immunity-related signaling protein kinases. These DEGs are listed in Supplementary Tables 16–20. It is to be noted that the phytohormone-related DEGs presented in this Figure are involved in signaling instead of synthesis or degradation.

Plants possess an enormous number of cell-surface localized and intracellular receptors to perceive early immunogenic signals, containing pattern recognition receptors (PRRs, namely, leucine-rich repeat receptor-like proteins and kinases) and nucleotide-binding–leucine-rich-repeat (NLR) receptors consisting of toll/interleukin-1 receptor/resistance (TIR) and coiled-coil (CC) types (Monteiro and Nishimura, 2018; Wan et al., 2019). There were 41, 60, and 38 DEGs related to PRRs in HLB_B/L/R, respectively (Supplementary Table 14). The majority of PRRs were upregulated in HLB_B/R but were downregulated in HLB_L (Figure 6). Surprisingly, two well-known PRRs, *flagellin-sensing 2* (*FLS2*) and *EF-Tu receptor* (*EFR*), were identified as DEGs in HLB_L/R but not in HLB_B (Supplementary Table 14). As for NLRs, only one CC-type NLR was found in HLB_B, but 30, 23, and 12 TIR-type NLRs were identified in HLBs_B/L/R, respectively (Supplementary Table 15). All the NLRs were annotated as disease resistance proteins whose transcriptional expressions were all nearly upregulated in HLB_B, but half of them were downregulated in HLB_L (Figure 6). The transcriptional expressions of other upstream immunity-related signaling protein kinases, such ascysteine-rich receptor-like kinases (CRKs), glutamate-like receptors (GLRs), mitogen-activated protein kinase kinase kinases (MAPKKKs), and mitogen-activated protein kinases (MAPKs), were also significantly changed in HLB_L/B/R but varied among the tissues (Figure 6). Calcium- and phytohormone-mediated signal transduction was mentioned in the following parts.

### DEGs Involved in Plant-Pathogen Interaction and Plant Hormone Signal Transduction

In the “plant-pathogen interaction” pathway, there were more GMEs in the *C*Las-infected tissues (Figure 5C), indicating that *C*Las infection triggered the innate immunity system to fight against the pathogen. In this pathway, there were 31% GMEs in HLB_B (6% in CY_B), 27% in HLB_L (8% in CY_L), and 15% in HLB_R (12% in CY_R). These findings suggested that HLB might cause more defense responses in barks and leaves than in roots after 18 weeks of inoculation. There were 30, 36, and 9 DEGs related to plant-pathogen interaction enriched in the barks, leaves, and roots, respectively (Supplementary Table 16). These DEGs included *calmodulin-like proteins* (*CML3, 8, 16, 18, 23, 25, 27, 30, 38, 41, 42*, and *44*), *calcium-dependent protein kinases* (*CPK2, CPK5*, and *CPK9*), *plant respiratory burst oxidase homologs* (*RBOHB, RBOHC, RBOHD*, and *RBOHF*), *cyclic nucleotide-gated ion channels* (*CNGC1, CNGC2*, and *CNGC8*), *enhanced disease susceptibility 1* (*EDS1*), *heat shock protein 90s* (*HSP90-1* and *HSP90-5*), *disease resistance proteins* (*RIN4, RPM1*, and *RPP13*), *WRKYs* (*WRKY22* and *WRKY33*), etc.

In the “plant hormone signal transduction” pathway, the number of GMEs increased from 7 to 21% in the barks and 15 to 26% in the leaves, but it decreased from 19 to 12% in the roots after *C*Las infection (Figure 5C). There were 31, 42, and 10 DEGs in the barks, leaves and roots, respectively (Supplementary Table 17), including four DEGs mainly related to abscisic acid (ABA), 28 related to auxin, 11 related to cytokinin, five related to ethylene, three related to gibberellin (GA), eight related to jasmonic acid (JA), and seven related to salicylic acid (SA). Among these DEGs, there were genes encoding kinases, such as *Arabidopsis histidine kinase 4* and *ethylene receptor 2*. Some DEGs encoded transcription factors, such as *ethylene insensitive 3* and *TGACG-binding factors* (*TGAs*). Several *non-expresser of pathogenesis-related genes* (*NPR*) were also identified, such as *NPR3* and *NPR5*. Interestingly, all the five DEGs related to ethylene were universally upregulated and exclusively found in the *C*Las-infected leaves. Although the DEGs related to SA were mainly detected in the *C*Las-infected leaves, five out of the six were downregulated. Moreover, all the DEGs related to JA were upregulated regardless of tissue type. Expressions of some DEGs involved in plant-pathogen interaction and plant hormone signal transduction are illustrated in Figure 6.

### HLB Affected the Accumulation of Transcripts and Metabolites Related to Carbohydrate Metabolism

**Figure 7:**
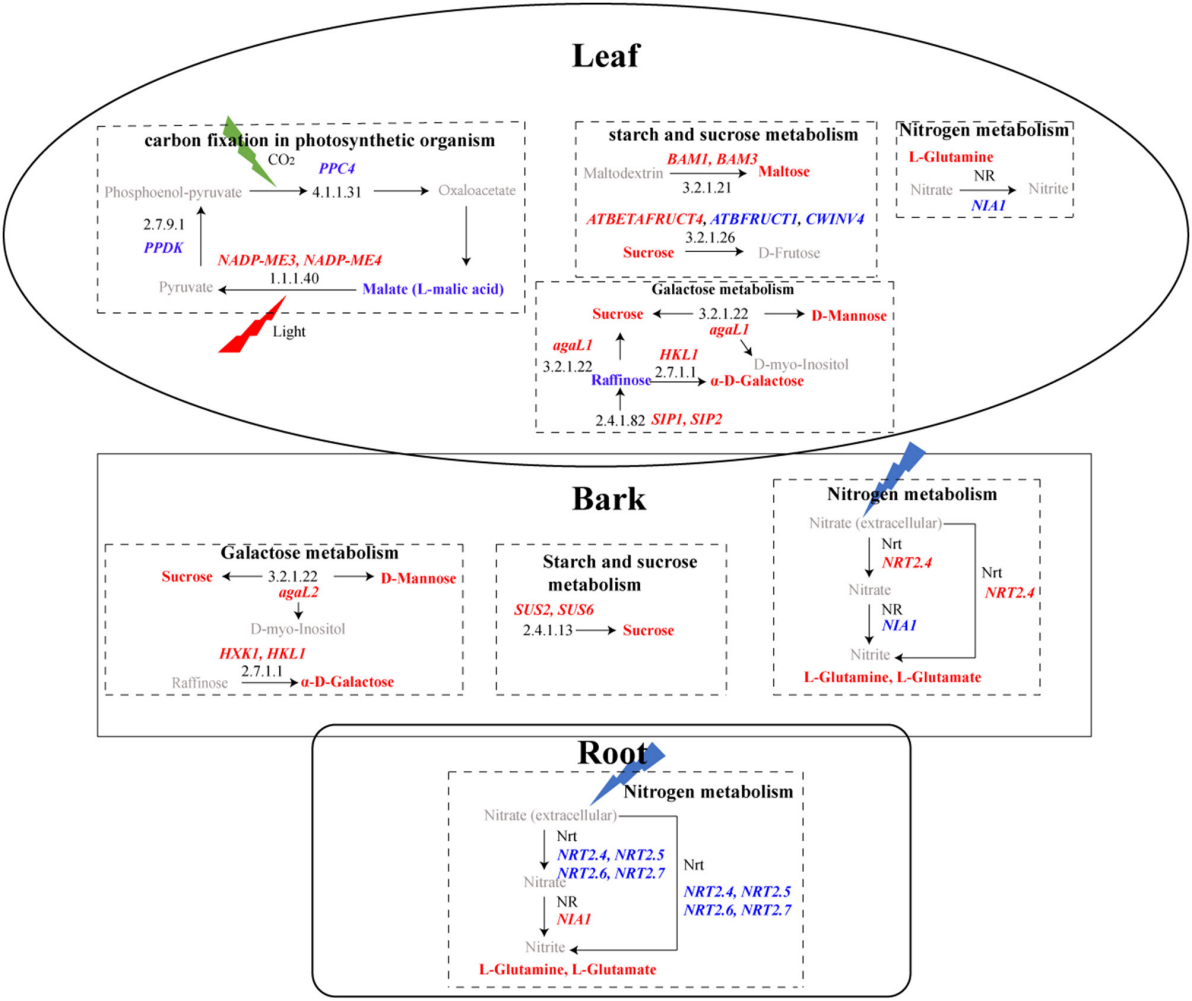
KEGG pathways with DEGs and DAMs in leaves, bark, and roots. In the rectangular box, pathways are represented in bold black, and black numbers represent the KEGG ID of enzymes. Upregulated genes are in red bold italicized words, while downregulated genes are in blue bold italicized words. The red bold represents upregulated metabolites, while the blue bold represents downregulated metabolites. Gray represents metabolites that were not detected.

At the transcriptional level, a total of 74 DEGs in “starch and sucrose metabolism” and 23 in “galactose metabolism” were detected (Supplementary Table 18), and more GMEs in HLB_L, as well as HLB_B, were observed in these two pathways (Figure 5C). Some of the DEGs were suggested to be involved in cell wall modification, and the majority of their expressions were upregulated in HLB_L/B (Figure 6). Integrated transcriptomics and metabolomics results revealed that these two pathways were enriched in both the leaves and the barks with the detection of related DEGs and DAMs, but that no related DAMs were detected in the roots (Figure 7). To be specific, significantly increased sucrose and maltose contents in HLB_L were accompanied by the downregulation of two *cell wall invertase* (*CWINV*), β-*fructofuranosidase 1* (*ATBFRUCT1*) and *CWINV4*, and by the up-regulation of *vacuolar invertase, At-*β*-Fructosidase4* (*ATBETAFRUCT4*), β*-amylase1* (*BAM1*), and *BAM3*in “starch and sucrose metabolism”. In the “galactose metabolism” of HLB_L, the contents of sucrose, D-mannose, D-myo-inositol, α-D-galactose, and raffinose were all elevated, and so were the transcripts of α*-galactosidase1* (*agaL1*), *hexokinase-like 1* (*HKL1*), *seed imbibition protein 1/2* (*SIP1/2*, genes encoding raffinose synthase. In the barks, the upregulation of *sucrose synthase2* (*SUS2*), *SUS6*, and *agaL2* promoted sucrose production. The upregulation of*HKL1* and *hexokinase 1* (*HXK1*) elevated the accumulation of α-D-galactose (Figure 7).

### HLB Altered the Accumulation of Photosynthesis-Related Transcripts and Metabolites

Two photosynthesis-related KEGG pathways were enriched: “photosynthesis-antenna protein” and “carbon fixation in photosynthetic organism”. The genes related to photosynthesis-antenna protein all belonged to the light-harvesting chlorophyll a/b-binding protein (LHCP) family (Supplementary Table 19). These 12 *LHCPs* only reached their maximum expression in the healthy leaves (Figure 5C) and were universally downregulated in the *C*Las-infected barks (Figure 6). Because their fragments per kilobase of exon model per million mapped fragments in the *C*Las-infected leaves/barks/roots were 788, 211, and 1, respectively, these results indicated that photosynthesis mainly occurred in the leaves and was hampered by HLB.

There were 18 DEGs enriched in “carbon fixation in photosynthetic organism” (Supplementary Table 19) and more GMEs in the healthy leaves and barks (Figure 5C). Their expressions were downregulated in the *C*Las-infected tissues (Figure 6). Although the malate content in HLB_L was significantly higher and two *NADP-dependent malic enzymes* (*NADP-ME3/4*) were upregulated, the downregulation of *phosphoenolpyruvate carboxylase 4*(*PPC4*) and *Pyruvate orthophcosphate dikinase* (*PPDK*) may significantly reduce CO_2_ fixation in the leaves (Figure 7). Therefore, photosynthesis was severely impaired in the *C*Las-infected leaves.

### HLB Regulated DEGs and DAMs Involved in Carotenoid Biosynthesis and Nitrogen Metabolism

Carotenoids are essential for physiological processes in plants, such as photosynthesis and the production of phytohormones (Cazzonelli and Pogson, 2010). The number of GMEs in the healthy and *C*Las-infected barks was the same, but it decreased from 40 to 7% in the roots after infection (Figure 5C). Moreover, DEGs encoding carotenoid cleavage dioxygenases (CCDs), phytoene synthase (PSY), Zeaxanthin epoxidase (ZEP), and the iron-binding protein D27 were all downregulated in HLB_R (Supplementary Table 20). Thus, carotenoid biosynthesis was negatively influenced by HLB in the roots (Figure 6).

Nitrogen metabolism was the only pathway with detection of both DEGs and DAMs in the three tissues (Figure 7). Even though there were no DEGs directly related to it, L-glutamine was over-accumulated in all the three *C*Las-infected tissues. Among the DEGs related to nitrogen metabolism (Supplementary Table 20), five *high-affinity nitrate transporters* (*NRTs*) were all downregulated in HLB_R (Figure 6), which might influence the absorption of nitrogen from the external environment to the plant.

### Putative Marker Genes in Tissues for Diagnosis of HLB Infection

By scanning tau values among all the tissues, a total of 26 genes that had tau > 0.85 in the healthy tissues and reached their maximal expression in the corresponding *C*Las-infected tissues were considered as putative markers of HLB infection, including 13 genes in the leaves, 11 in the barks, and two in the roots (Supplementary Table 21).

**Figure 8:**
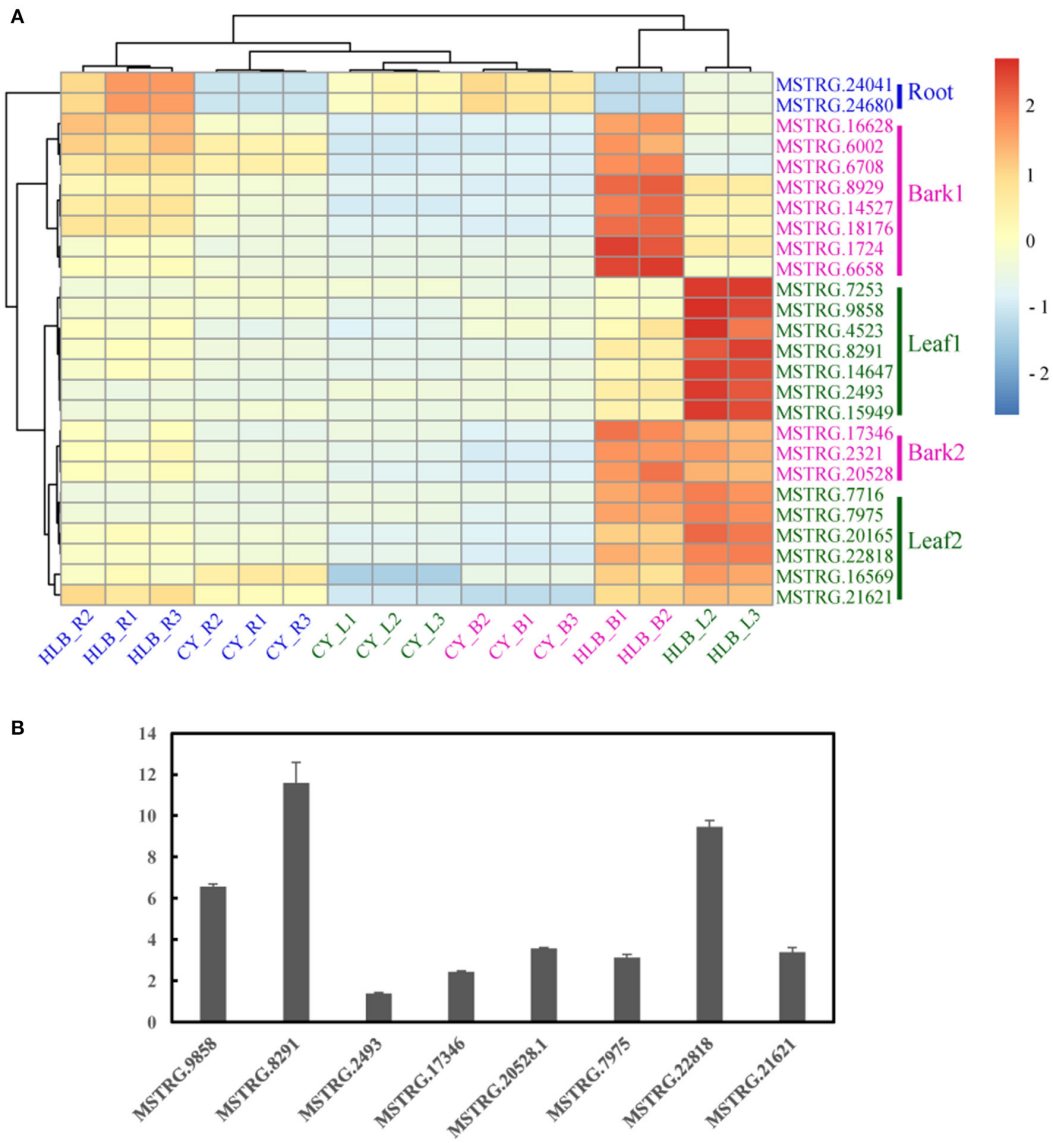
Heatmap of 26 putative marker genes in the three tissues **(A)** and quantitative reverse transcription (qRT)-polymerase chain reaction (PCR) verification of several putative marker genes in the leaves. **(B)** The expression level of the putative marker gene in healthy leaves was set as 1 in the qRT-PCR analysis.

Based on the expression patterns of these 26 putative markers, the samples were vertically divided into two main clusters: one containing *C*Las-infected roots and all the controls, and the other containing *C*Las-infected barks and leaves (Figure 8A). Horizontally, cluster 1 contained two markers in Root; cluster 2 included eight markers in Bark 1; cluster 3 consisted of seven in Leaf 1, three in Bark 2, and six in Leaf 2. Therefore, genes in Root, Bark 1, and Leaf 1 could be considered as specific markers in the roots, barks and leaves, respectively. In addition, genes in Bark 2 and Leaf 2 could be putative gene makers in both *C*Las-infected barks and leaves due to their similar expression patterns.

Marker genes in Leaf 1, Bark 2, and Leaf 2 were verified by qRT-PCR using *C*Las-infected and healthy leaf samples from *C. sinensis*. Only eight putative marker genes could be consistently amplified, and their expression all increased in the *C*Las-infected samples (Figure 8B).

## Discussion

### Signal Perception and Propagation

Plants innately evolve a two-layered immune system, consisting of pattern-triggered immunity (PTI) and effector-triggered immunity (ETI) (Zhou and Zhang, 2020). The activation of the first layer of plant immunity PTI is initiated by intercellular receptors named PRRs, and the second layer, ETI, is conditioned by intracellular receptors named NLRs (Wan et al., 2019). NLRs contain TIR-type and CC-type NLRs depending on N-terminal singaling domains (Monteiro and Nishimura, 2018). In our study, many PRRs and NLRs were differentially expressed in the *C*Las-infected tissues (Supplementary Table 14; Figure 6), such as the two best characterized PRRs: flagellin-sensing 2 (FLS2) and EF-Tu receptor (EFR). Consistent with a recent report that PTI and ETI mutually potentiate to activate strong defenses against pathogens (Ngou et al., 2021), the majority of PRRs and NLRs in HLB_B/R were upregulated, but those in HLB_L were downregulated (Figure 6). A critical early signaling event connecting PTI and ETI is the production of reactive oxygen species (ROS) by NADPH oxidase respiratory burst oxidase homologs (RBOHs), such as RBOHB/D (Adachi et al., 2015; Yuan et al., 2021). However, the phosphorylation of WRKYs, essential components of both PTI and ETI, by MAPK is required for RBOHs-mediated ROS bursts (Adachi et al., 2015; Ngou et al., 2021). In addition, RBOHD could be directly activated by cysteine-rich receptor-like kinase 2 (CRK2) by phosphorylation, and the *crk2* mutant was impaired in the full elicitor-induced ROS bursts as well as defense against pathogen attack (Kimura et al., 2020). In the barks, nearly all the above-mentioned genes were upregulated after *C*Las inoculation (Figure 6). Although the majority of *CRKs* (especially *CRK2*) were downregulated in HLB_L/R, the up-regulation of *WRKYs, MAPKKKs*, and *MAPKs* may compensate for the activation of RBOHB/C/D, whose transcripts were all significantly increased in these two tissues (Figure 6; Supplementary Table 16).

Most of the DEGs encoding other upstream immunity-related signaling protein kinases, glutamate-like receptors (GLRs), were upregulated in all the HLB tissues (Figure 6), which were suggested to be involved in various physiological pathways, such as Ca^2+^ signaling (Grenzi et al., 2021). Ca^2+^ is the most prominent second messenger that functions in a wide variety of environmental responses and developmental processes in plants (Lee and Seo, 2021). EF-hand calcium-binding proteins play a principal role in calcium signaling in plants, such as calmodulin (CaM), cyclic nucleotide-gated channel (CNGC), calmodulin-binding protein (CML), ATPase E1-E2 protein, and calcium-dependent protein kinase (CPK) (Mohanta et al., 2019). One *CaM* and three *CNGCs* were identified as DEGs in our study (Supplementary Table 16), which were reported to be critical in linking pathogen patterns to plant immunity (Tian et al., 2019). In total, 10 out of the 13 *CMLs* were upregulated (Supplementary Table 16), further proving their close relationship with plant defense (Lv et al., 2019). CPKs are calcium sensors and play important roles in ETI. For instance, *CPK5* overexpressing *Arabidopsis* exhibited TN2-dependent autoimmunity and enhanced disease resistance (Liu et al., 2017). Meanwhile, CPK5 directly phosphorylated RBOHD *in vivo*, which was critical for rapid defense signal transduction and ROS-mediated cell-to-cell communication (Dubiella et al., 2013), consistent with the upregulation of *CPK5* and *RBOHD* in HLB_L/B (Supplementary Table 16). In addition, ATPase E1-E2 protein, whose transcript significantly accumulated in HLB_L/B, was involved in Ca^2+^-translocation (Hayter and Peterson, 2004) (Supplementary Tables 2, 3). Since more than half of the DEGs identified in the plant-pathogen interaction pathway were calcium-related (Supplementary Table 16), calcium-based defense signal propagation is probably a pivotal event in *C*Las infection.

### Multi-Immune Responses

To prevent biotrophic pathogen invasion, plant cells would initiate immune responses, such as autophagy, the ubiquitin-proteasome system, and programmed cell death (Greenberg and Yao, 2004; Üstün et al., 2018). There was one upregulated DEG annotated as *autophagy-related protein* (*ATG*) in each *C*Las-infected tissue, namely, *ATG11* in the barks, *ATG18H* in the leaves and roots (Supplementary Tables 2–4). The ubiquitin-proteasome system contains various components, such as plant U-box (PUB) E3 ligases, FLS2, RBOHD (Trujillo, 2021), whose transcripts nearly all significantly accumulated in the *C*Las-infected tissues. These results suggested that HLB may trigger autophagy and the ubiquitin-proteasome system in Chongyi. In addition, transcripts of *hypersensitive-induced reaction* (*HIR*)*1* and *HIR2* significantly accumulated (Supplementary Table 2), which were reported to participate in the hypersensitive response (a programmed cell death phenomenon) and positively contributed to basal resistance against pathogen attack in the enhanced disease susceptibility 1 (EDS1)- and salicylic acid (SA)-dependent pathways (Qi et al., 2011; Li et al., 2019). Meanwhile, HIRs could contribute to ETI by forming complexes with a membrane-associated disease resistance protein RPS2 (Qi et al., 2011), whose transcriptional expression was also upregulated in the *C*Las-infected barks (Supplementary Table 2). Thus, multi-immune responses were triggered.

The EDS1 family of immunity regulators contains three proteins: EDS1, phytoalexin deficient 4 (PAD4), and senescence-associated gene 101 (SAG101), which could form mutually exclusive EDS1-SAG101 and EDS1-PAD4 heterodimers (Ngou et al., 2021). EDS1-SAG101 dimers have a helper NLR to mediate cell death and pathogen resistance (Lapin et al., 2019). EDS1-PAD4 functions as an integrator of cell surface and intracellular receptor signaling, which delivers signals from TIR-NLRs to transcriptional defense and host cell death (Dongus and Parker, 2021). Meanwhile, the EDS1-PAD4 module participates in phytohormone-mediated pathogen containment by inhibiting MYC2, a negative regulator of SA biosynthesis, and enhances the SA defense sector (Cui et al., 2018). Therefore, EDS1 is considered an immune regulatory hub (Lapin et al., 2020). In our study, *EDS1* and *PAD4* were both upregulated in the *C*Las-infected leaves and barks. Although *SAG101* was not found, its two homologs were identified as DEGs, and the upregulation of *SAG20* and *SAG21* was consistent with their induced expression upon pathogen attack (Keates et al., 2003; Salleh et al., 2012).

### Phytohormone Signal Pathways and Defense Response

Phytohormones, such as SA, jasmonic acid (JA), ethylene (ET), abscisic acid (ABA), auxin, and gibberellins (GAs), participate in defense responses (Berens et al., 2017). Among them, SA and JA are major defense-related phytohormones, while the other phytohormones participate in hormone signaling mainly through interactions with SA and JA (Pieterse et al., 2009). The transcriptional levels of DEGs involved in plant hormone signal transduction varied among the *C*Las-infected tissues, especially those related to SA (Supplementary Table 17; Figure 6). Non-expressor of pathogenesis-related genes 1 (NPR1), the main protein required for SA perception, interacts with TGACG-binding (TGA) transcription factors, which modulate SA biosynthesis positively (Budimir et al., 2021). Although *NPR1* was not found in our study, *Blade-on-petiole 2* (*BOP2*, a paralog of *NPR1*) and *TGA1/7/9* were all downregulated in the *C*Las-infected leaves. Meanwhile, *Cs2g10790* (*NPR3*), whose protein transcriptionally repressed genes involved in promoting SA-mediated basal immunity, such as *systemic acquired resistance deficient 1* (*SARD1*) and *WRKY70*, was upregulated in the *C*Las-infected leaves and barks (Supplementary Table 17) (Ding et al., 2018). However, *SARD1* and over 80% of the *WRKYs* were upregulated in HLB_L/B, probably because of the compensatory mechanism(s) to circumvent weakened SA signaling, such as MAPK signaling (Tsuda et al., 2013). In addition, three DEGs annotated as *phenylalanine ammonium-lyase 1* (*PAL1*), encoding the key enzyme of the PAL pathway contributing to SA biosynthesis in immunity (Berens et al., 2017), were upregulated in HLB_B (Supplementary Table 2). Therefore, the above information indicated that SA-mediated defense response might be partially depressed in the leaves but not in the barks after *C*Las inoculation.

The pathogen of citrus canker appeared to promote JA accumulation through hijacking host ABA production, which could suppress SA-mediated defenses and benefit pathogen development (Long et al., 2019). Consistently, DEGs involved in JA, ET, and ABA signaling were nearly all upregulated in the *C*Las-infected leaves and barks (Figure 6), and so were those participating in JA biosynthesis, such as *allene oxide cyclase 4* (*AOC4*), *lipoxygenase 3*(*LOX3*), *12-oxophytodienoate reductase 2*(*OPR2*), and *peroxisomal acyl-coenzyme A oxidase 1* (*ACX1*, Supplementary Tables 2, 3). Hence, JA signaling was probably dominated in the *C*Las-infected leaves and barks, which may suppress SA-mediated pathogen defense.

### Alteration of Carbohydrate Metabolism and Cell Wall Composition

Huanglongbing severely influences functions related to carbohydrate biosynthesis and metabolism (Wu et al., 2020). The swelling of the middle lamella among cell walls surrounding sieve elements would drive starch accumulation in the leaves and phloem, and decrease starch transport to roots (Folimonova and Achor, 2010). In this study, the “starch and sucrose metabolism” and “galactose metabolism” pathways were upregulated in the *C*Las-infected leaves and barks but were down-regulated in the roots (Figure 5C). Consistently, the metabolomics results revealed that sucrose accumulation occurred in both the *C*Las-infected leaves and barks, but that no DAMs were detected in the roots (Figure 7), which might result in low carbohydrate storage in the roots.

Interestingly, many carbohydrate-related DEGs were annotated to participate in cell wall modification and remodeling. Cellulose, various hemicelluloses, and pectins are the three major types of polysaccharides in the cell wall of Arabidopsis leaves (Liepman et al., 2010). CEL1, an endo-1,4-β-glucanase, was required for cellulose formation of the cell wall during cell growth and expansion (Tsabary et al., 2003), and its transcripts significantly accumulated in the *C*Las-infected barks and roots (Supplementary Table 18). UDP-D-glucuronate 4-epimerases (GAEs) provide important precursors for the synthesis of pectins, which are the major component of the plant primary cell wall and critical for its integrity as well as immunity (Mølhøj et al., 2004; Bethke et al., 2016). *GAE1/6* were depressed upon *Botrytis cinerea* and *Pma* ES4326 challenge in Arabidopsis (Bethke et al., 2016), but they were induced in our study (Supplementary Table 18). Meanwhile, several DEGs encoding pectin lyase-like superfamily proteins, which were reported to participate in plant cell wall modification (Hocq et al., 2020), were also identified (Supplementary Table 18). Pectinesterases (PMEs) contribute to cell wall integrity (Bethke et al., 2014), which may explain the upregulation of 11 *PMEs* (Supplementary Table 18).

UDP-glucose 4-epimerase (UGE) provides UDP-galactose for cell wall biosynthesis (Rösti et al., 2007). UDP-glucose 6-dehydrogenases 2 (UGD2) and UGD3 were required for the formation of the cell wall in growth (Reboul et al., 2011). Inferred from electronic annotation on the website of UniProtKB (https://www.uniprot.org/uniprot/), hexosyltransferases (GAUTs) may be involved in pectin and/or xylans biosynthesis in cell walls, and alpha-galactosidase 1/2 (AGAL1/2) may function in cell wall loosening and expansion. The expression of *UGE1, UGD2, UGD3, GAUTs*, and *AGAL1/2* was all significantly upregulated (Supplementary Table 18). Meanwhile, trehalose was suggested to play a role in cell wall modification (Bae et al., 2005). Its biosynthesis in plants needs two indispensable enzymes: trehalose-phosphate synthase (TPS) and trehalose-6-phosphate phosphatase (TPP). The *tps1* mutant in *Arabidopsis* showed an altered cell wall structure and starch accumulation (Gómez et al., 2006), suggesting that the downregulation of two *TPS1* genes in the *C*Las-infected leaves may also cause starch accumulation. Therefore, cell wall was highly affected in Chongyi after *C*Las infection, which was considered as one of the typical characteristics of HLB-susceptible genotypes (Curtolo et al., 2020).

### HLB Negatively Affects Carotenoid and Nitrogen Metabolism in Root

Carotenoids play essential roles in plant physiological processes, and phytoene synthase (PSY) is considered the most important regulatory enzyme in carotenogenesis (Cazzonelli and Pogson, 2010). *PSY* was suppressed in the *C*Las-infected roots (Supplementary Table 20), and so was *zeaxanthin epoxidase* (*ZEP*), encoding the other enzyme of carotenoid biosynthesis. Carotenoid cleavage dioxygenases (CCDs) can catalyze carotenoids into various carotenoid cleavage products and provide substrates for phytohormone biosynthesis (Gao et al., 2021). *CCD7* and *CCD8* were all downregulated in the roots after *C*Las infection (Supplementary Table 20), probably because the suppression of carotenoid biosynthesis resulted in the reduction of degradation. Interestingly, strigolactones are carotenoid-derived phytohormones, whose biosynthesis requires CCD7 and CCD8 as well as the iron-binding protein D27 (Alder et al., 2012; Wang et al., 2020). *D27* was down-regulated in *C*Las-infected roots, too. Consequently, HLB may repress strigolactone production in roots. However, root secrets strigolactones into the rhizosphere as a pre-symbiotic signal for arbuscular mycorrhizal symbiosis, which is critical for *Citrus* to obtain essential nutrients from the soil (Akiyama et al., 2005; An et al., 2018). Therefore, the suppression of carotenoid metabolism may cause a nutrient deficiency in roots, such as nitrogen (N) deficiency. Consistently, *high-affinity nitrate transports* (*NRTs*) *2.4, NRT2.5, NRT2.6*, and *NRT2.7*, which could reduce the absorption of N from soil to roots, were all downregulated (Figure 7; Supplementary Table 20). Thus, the suppression of carotenoid and nitrogen metabolism may accelerate the deterioration of the *C*Las-infected roots and weaken the defense response of the whole plant.

### A Hypothesized Model for HLB Susceptibility Mechanism in Chongyi Wild Mandarin

**Figure 9:**
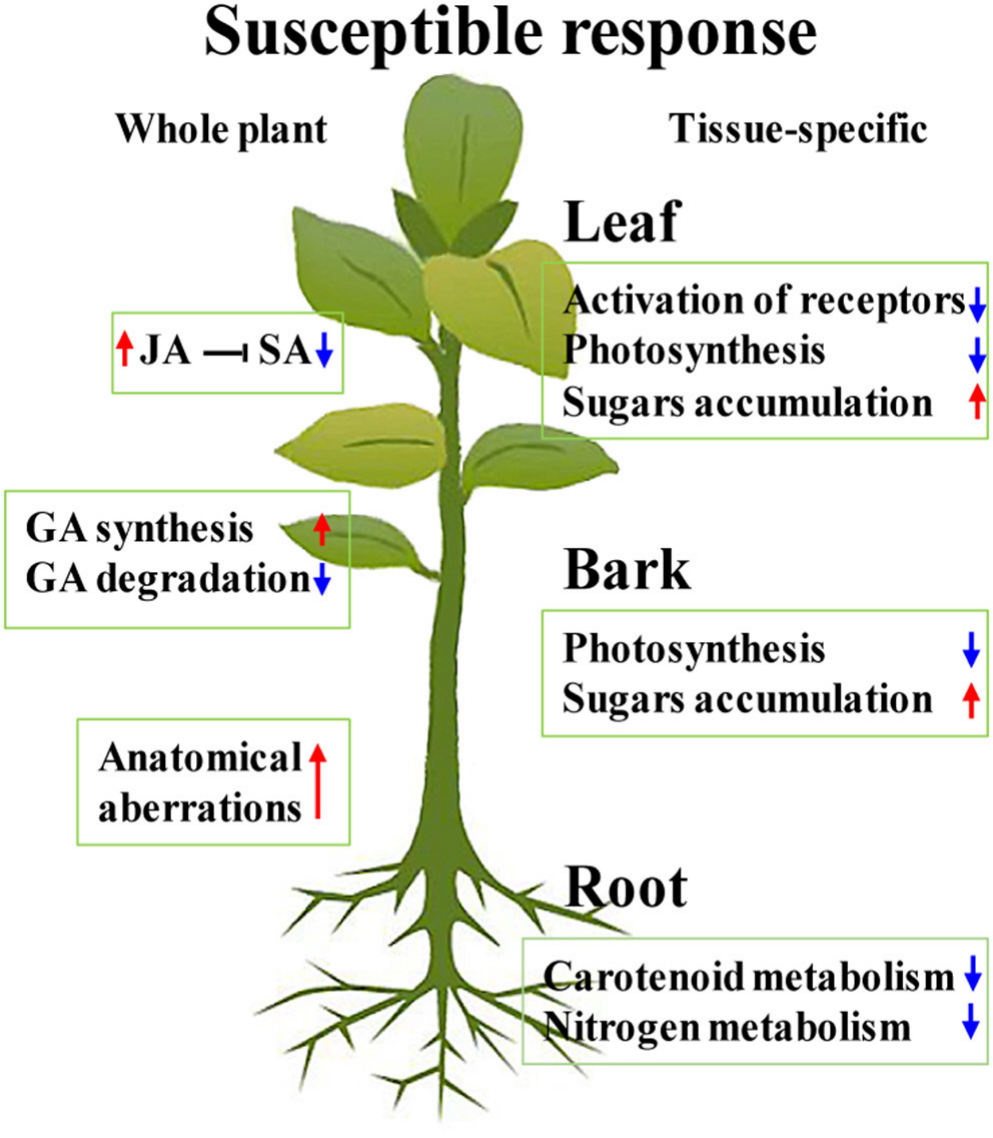
Hypothesized model for the whole plant and tissue-specific HLB susceptible response of Chongyi. Red arrows indicate induction, and blue ones indicate repression.

The comparison of transcriptomic profiles from the *C*Las-susceptible citrus genotypes and *C*Las-tolerant/resistant genotypes revealed the transcriptional alterations of susceptible plants, such as downregulation of receptors and WKRYs, induction of GA synthesis, and suppression of GA degradation (Curtolo et al., 2020). As mentioned above, the majority of receptors were depressed in the *C*Las-infected leaves (Figure 6). Although most *WRKYs* were upregulated in our study (Figure 6), *WRKY70*, which was suggested to play an important role in *Poncirus trifoliata* tolerance to HLB, was excluded (Peng et al., 2020). Two *gibberellin 2-beta-dioxygenase 8* genes in the *C*Las-infected leaves and one, which was involved in GA degradation, in the barks were downregulated (Supplementary Tables 2, 3) (Liu et al., 2021). No GA synthesis-related DEGs were identified, but several *Gibberellin stimulated Arabidopsis genes* (*GASAs*) were upregulated in all the *C*Las-infected tissues (Supplementary Tables 2–4). *GASAs* were GA-induced and could promote GA response (Rubinovich and Weiss, 2010), suggesting that GA synthesis was probably induced in *C*Las-infected Chongyi. Thus, these results were consistent with the characteristics of susceptible *Citrus* genotypes, as summarized by Curtolo et al. (2020). Furthermore, *C*Las-enhanced JA synthesis and signaling may hamper defense response by antagonizing SA, making Chongyi more vulnerable to HLB. Based on the above arguments, along with severe anatomical aberrations in the *C*Las-infected tissues, a hypothesized model of the whole plant and tissue-specific HLB susceptible response of Chongyi is illustrated in Figure 9.

## Data Availability Statement

The datasets presented in this study can be found in online repositories. The names of the repository/repositories and accession number(s) can be found in the article/Supplementary Material.

## Author Contributions

TP and B-LZ conceived the study. X-TX, F-TC, and X-JZ conducted the management of grafting and seedling. TP and J-LK contributed to data analysis and writing of the manuscript. W-SD, MW, Z-YL, and H-NS significantly contributed to the writing of the manuscript. All authors discussed the results and commented on the manuscript.

## Funding

This study was supported by the Natural Science Foundation of Jiangxi Province (20152ACB21005), the National Natural Science Foundation of China (32060670), the Major Science and Technology R&D Program of Jiangxi Province (20194ABC28007), and the National Key Research and Development Program of China (2018YFD0201500).

## Conflict of Interest

The authors declare that the research was conducted in the absence of any commercial or financial relationships that could be construed as a potential conflict of interest.

## Publisher's Note

All claims expressed in this article are solely those of the authors and do not necessarily represent those of their affiliated organizations, or those of the publisher, the editors and the reviewers. Any product that may be evaluated in this article, or claim that may be made by its manufacturer, is not guaranteed or endorsed by the publisher.

## Supplementary Material

Supplementary Figure 1 Detection of *C*Las in seedlings grafted with **(a)** healthy and **(b)** *C*Las-positive buds. M, marker; P, positive control; N, negative control.

Supplementary Figure 2 Heatmap of all metabolites in the three tissues.

Supplementary Figure 3 **(a)** Venn diagram of DEGs among leaves, bark, and roots, and **(b)** K-mean analysis results of common DEGs expression patterns to choose best cluster number.

Supplementary Figure 4 Expression patterns of six common DEGs among three comparisons in “starch and sucrose metabolism”.

Supplementary Table 1 Quality estimation of raw reads in all transcriptome.

Supplementary Table 2 DEGs of Clas-infected barks and healthy barks.

Supplementary Table 3 DEGs of Clas-infected leaves and healthy leaves.

Supplementary Table 4 DEGs of Clas-infected roots and healthy roots.

Supplementary Table 5 KEGG pathway enrichment of DEGs between Clas-infected and healthy barks.

Supplementary Table 6 KEGG pathway enrichment of DEGs between Clas-infected and healthy leaves.

Supplementary Table 7 KEGG pathway enrichment of DEGs between Clas-infected and healthy roots.

Supplementary Table 8 KEGG pathway enrichment of DAMs between Clas-infected and healthy barks.

Supplementary Table 9 KEGG pathway enrichment of DAMs between Clas-infected and healthy leaves.

Supplementary Table 10 KEGG pathway enrichment of DAMs between Clas-infected and healthy roots.

Supplementary Table 11 The 56 Common KEGG pathways enriched by DAMs and DEGs in bark.

Supplementary Table 12 The 54 common KEGG pathways enriched by DAMs and DEGs in leaf.

Supplementary Table 13 The 45 common KEGG pathways enriched by DAMs and DEGs in root.

Supplementary Table 14 DEGs related to leucine-rich repeat receptor-like protein kinase in bark, leaf and root.

Supplementary Table 15 DEGs related to TIR-NBS-LRRs or CC-NBS-LRRs in bark, leaf and root.

Supplementary Table 16 DEGs related to plant-pathogen interaction.

Supplementary Table 17 DEGs related to plant hormone signal transduction.

Supplementary Table 18 DEGs related to starch, sucrose and galactose metabolism.

Supplementary Table 19 DEGs related to carbon fixation and photosynthesis-antenna proteins.

Supplementary Table 20 DEGs related to carotenoid biosynthesis and nitrogen metabolism.

Supplementary Table 21 Fifteen Putative gene markers of CLas in leaves, barks and roots. Tau healthy values are represented as the tau values of healthy leaves, barks and roots, and tau symptomatic values are represented as the tau values of CLasinfected leaves, barks and roots.
