# Supplementary figures and images for: Integrated Transcriptomics and Metabolomics Analyses Provide Insights Into the Response of Chongyi Wild Mandarin to Candidatus Liberibacter Asiaticus Infection
Source: Front Plant Sci. 2021 Oct 14;12:748209. doi: 10.3389/fpls.2021.748209 (PMC8551615; doi:10.3389/fpls.2021.748209)

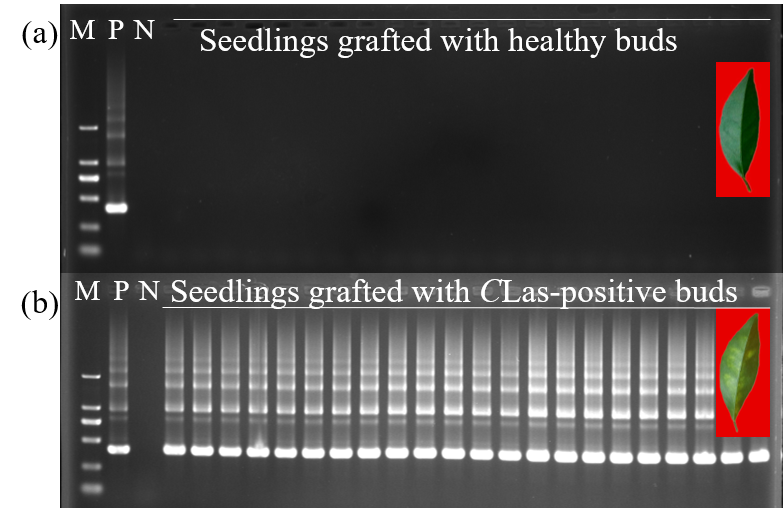

Supplement: Supplementary Figure 1 — Detection of CLas in seedlings grafted with (a) healthy and (b) CLas-positive buds. M, marker; P, positive control; N, negative control. [file Image_1.tif]

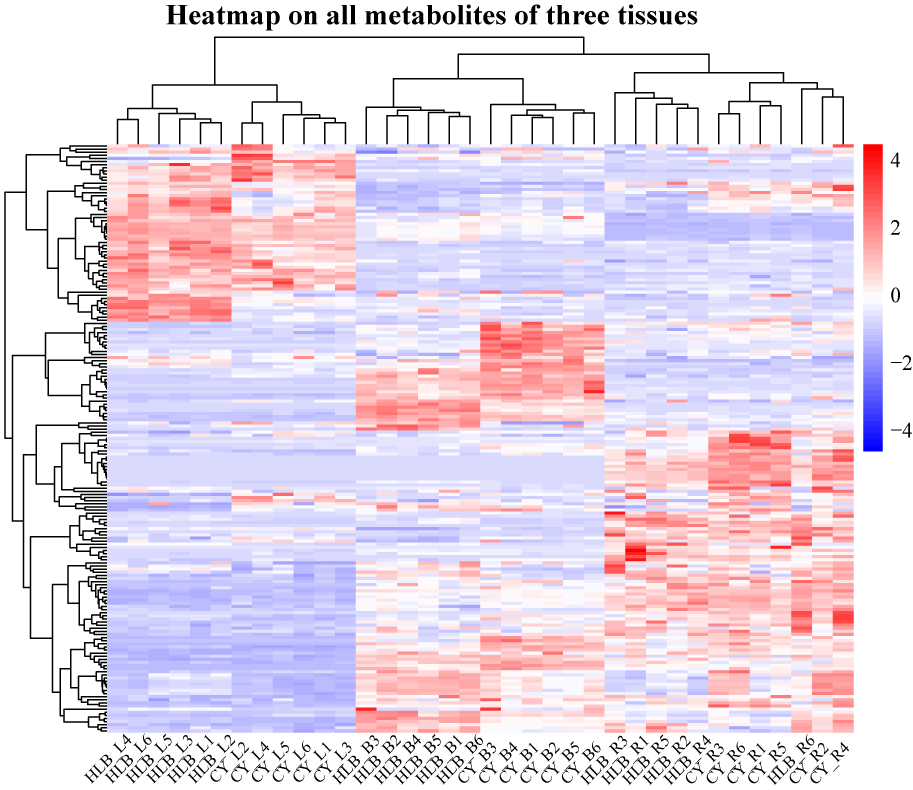

Supplement: Supplementary Figure 2 — Heatmap of all metabolites in the three tissues. [file Image_2.tif]

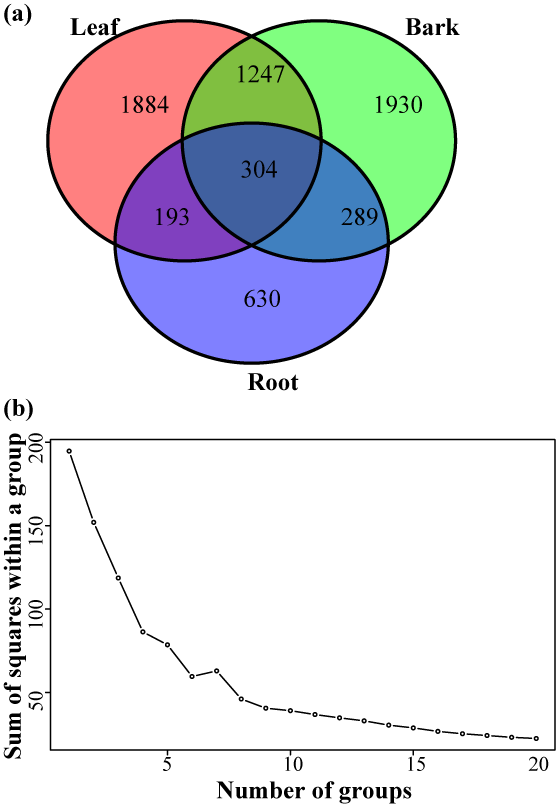

Supplement: Supplementary Figure 3 — (a) Venn diagram of DEGs among leaves, bark, and roots, and (b) K-mean analysis results of common DEGs expression patterns to choose best cluster number. [file Image_3.tif]

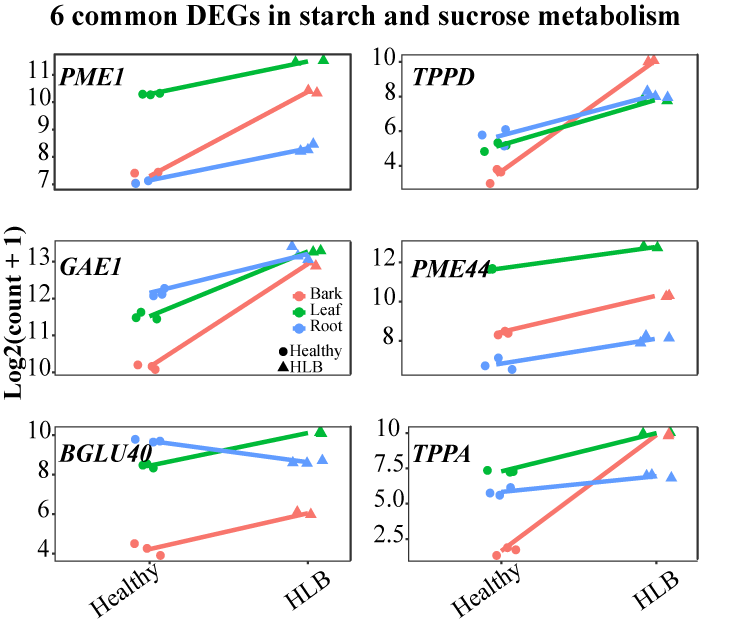

Supplement: Supplementary Figure 4 — Expression patterns of six common DEGs among three comparisons in “starch and sucrose metabolism”. [file Image_4.tif]
